# Degradation of NLRP3 by p62‐dependent‐autophagy improves cognitive function in Alzheimer's disease by maintaining the phagocytic function of microglia

**DOI:** 10.1111/cns.14219

**Published:** 2023-04-18

**Authors:** Dongyuan Zhang, Yu Zhang, Jirong Pan, Jingjing Cao, Xiuping Sun, Xianglei Li, Ling Zhang, Chuan Qin

**Affiliations:** ^1^ NHC Key Laboratory of Human Disease Comparative Medicine Beijing Engineering Research Center for Experimental Animal Models of Human Critical Diseases National center of Technology Innovation for animal model Changping National laboratory (CPNL) Institute of Laboratory Animal Sciences, Chinese Academy of Medical Sciences (CAMS) & Peking Union Medical College (PUMC) Beijing China

**Keywords:** Alzheimer's disease, autophagy, NLRP3 inflammasome, pyroptosis, sqstm1/p62, ubiquitin

## Abstract

**Background:**

Activation of the NLRP3 inflammasome promotes microglia to secrete inflammatory cytokines and induce pyroptosis, leading to impaired phagocytic and clearance functions of microglia in Alzheimer's disease (AD). This study found that the autophagy‐associated protein p62 interacts with NLRP3, which is the rate‐limiting protein of the NLRP3 inflammasome. Thus, we aimed to prove that the degradation of NLRP3 occurs through the autophagy‐lysosome pathway (ALP) and also demonstrate its effects on the function of microglia and pathological changes in AD.

**Methods:**

The 5XFAD/NLRP3‐KO mouse model was established to study the effect of NLRP3 reduction on AD. Behavioral experiments were conducted to assess the cognitive function of the mice. In addition, immunohistochemistry was used to evaluate the deposition of Aβ plaques and morphological changes in microglia. BV2 cells treated with lipopolysaccharide (LPS) followed by Aβ1‐42 oligomers were used as in vitro AD inflammation models and transfected with lentivirus to regulate the expression of the target protein. The pro‐inflammatory status and function of BV2 cells were detected by flow cytometry and immunofluorescence (IF). Co‐immunoprecipitation, mass spectrometry, IF, Western blot (WB), quantitative real‐time PCR, and RNA‐seq analysis were used to elucidate the mechanisms of molecular regulation.

**Results:**

Cognitive function was improved in the 5XFAD/NLRP3‐KO mouse model by reducing the pro‐inflammatory response of microglia and maintaining the phagocytic and clearance function of microglia to the deposited Aβ plaque. The pro‐inflammatory function and pyroptosis of microglia were regulated by NLRP3 expression. Ubiquitinated NLRP3 can be recognized by p62 and degraded by ALP, slowing down the proinflammatory function and pyroptosis of microglia. The expression of autophagy pathway‐related proteins such as LC3B/A, p62 was increased in the AD model in vitro*.*

**Conclusions:**

P62 recognizes and binds to ubiquitin‐modified NLRP3. It plays a vital role in regulating the inflammatory response by participating in ALP‐associated NLRP3 protein degradation, which improves cognitive function in AD by reducing the pro‐inflammatory status and pyroptosis of microglia, thus maintaining its phagocytic function.

## Graphical abstract

P62 can recognize and bind ubiquitin‐modified NLRP3. It plays a vital role in regulating the inflammatory response by participating in the ALP‐associated NLRP3 protein degradation, which improves cognitive function in AD by reducing pro‐inflammatory status and pyroptosis of microglia, thus maintaining its phagocytosis function.

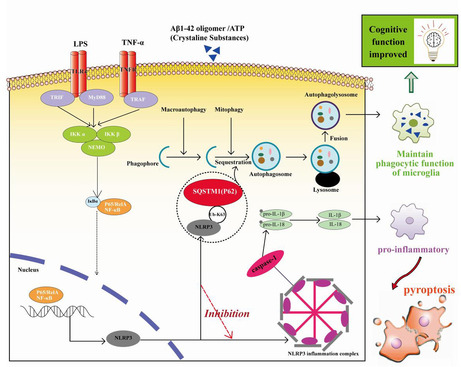

## 1 INTRODUCTION

Alzheimer's disease (AD) is a neurodegenerative disease that is characterized by progressive memory impairment, mental changes, and reduced social functionality.^1^, ^2^, ^3^ Since there is no effective treatment for AD, identifying effective treatment methods to improve AD progression is crucial. Among the different mechanisms of AD, a persistent and excessive inflammatory response is a common feature of AD which promotes pathological changes.^4^ Studies have indicated that the increased expression of NLRP3 in AD can aggravate pathological changes by enhancing the inflammatory response.^5^, ^6^, ^7^ However, how to regulate NLRP3 expression in AD remains unknown. Therefore, we focused on exploring the role of NLRP3 regulatory mechanisms in AD.

The NLRP3 inflammasome is present in the cytoplasm of microglia in the central nervous system. It contains apoptosis‐associated speck‐like protein containing CARD(ASC), pro‐caspase‐1, and NLRP3, the latter of these being the rate‐limiting protein in inflammasome formation.^8^ Microglia, which are believed to be of neuroectodermal origin,^9^ are a type of macrophage belonging to the immune system. Microglia can produce pro‐inflammatory or immunoregulatory mediators according to the phenotype of the polarization status, such as the M1 (classical activation) and M2 (alternative activation) phenotypes.^10^ However, changes in microglial phenotypes are complicated and may differ with the stage and severity of neurodegenerative diseases. Therefore, the M1/M2 nomenclature for microglia is oversimplified and cannot reflect the various microglial phenotypes.^11^, ^12^ Therefore, the use of M1/M2 was limited in this study, appearing only directly from the references where they were used.

Microglia play an important role in the biological processes of AD, such as plaque clearance and inflammatory responses. Under homeostatic conditions, microglia exhibit a highly ramified morphology with a small soma. Activated microglia can be classified into M1 polarization status with a pro‐inflammatory response^13^ and M2 polarization status with an anti‐inflammatory response and phagocytic function.^12^, ^14^, ^15^, ^16^ The M2 polarization status of microglia plays an important role in clearing Aβ plaques and alleviating neurotoxicity. However, as Nod‐like activators, Aβ plaques promote the synthesis and activation of the NLRP3 inflammasome, which ultimately leads to the secretion of inflammatory cytokines such as IL‐1β and IL‐18.^15^, ^16^, ^17^ Simultaneously, the activation of NLRP3 can induce pyroptosis in microglia. Ultimately, the inflammatory response and the lack of phagocytosis capacity in microglia lead to neuronal damage, exacerbating the progression of AD. Therefore, we aim to find a way to maintain the phagocytic function of microglia by reducing the expression of NLRP3 protein to inhibit the assembly and synthesis of NLRP3 inflammasomes.

This study identified dozens of proteins that can interact with NLRP3 and participate in its biological process by co‐immunoprecipitation (Co‐IP) combined with mass spectrometry analysis. Among these, p62, an autophagy‐related protein^18^ that recognizes ubiquitinated proteins or damaged organelles and participates in their degradation through the autophagy‐lysosomal pathway (ALP),^19^, ^20^, ^21^ aroused our interest. Hence, we hypothesized that ubiquitinated NLRP3 is recognized by p62 and transported to autophagosomes for degradation, which reduces the pro‐inflammatory response of microglia, maintains the phagocytic function of microglia, and alleviates the neuroinflammatory response and pathological changes in AD.

To test this hypothesis, we used 5XFAD mice to generate an NLRP3 knockout (KO) mouse model to investigate the effects of reduced NLRP3 expression on cognitive function and pathology in an Alzheimer's mouse model at different ages through behavioral, pathological, and molecular biological methods. Activation of the NLRP3 inflammasome is divided into two stages: priming and activation. Since LPS participates in the priming process by activating the TLR‐signaling molecule to initiate the NF–kB pathway^22^ and Aβ oligomers as activators to activate the expression of NLRP3 protein and the synthesis of the inflammasome, we used BV2 cells incubated with LPS followed by Aβ1‐42 oligomers to regulate the expression of NLRP3 and to simulate AD inflammation conditions.

The results showed that downregulating the expression of NLRP3 decreased microglial pro‐inflammatory effects and pyroptosis, increased the ability to clear Aβ plaques, and improved cognitive functions of the 5XFAD mice model in behavioral experiments such as new object recognition, the Morris water maze, and the Y maze. In addition, we showed that p62 recognized ubiquitin‐modified NLRP3, resulting in its degradation through the ALP, and inhibited the formation of NLRP3 inflammasomes, which affected microglial polarization. Autophagy occurs in AD in vitro models, as confirmed by RNA‐seq and Western Blot methods.

## 2 MATERIALS AND METHODS

### 2.1 Reagents

The drugs and antibodies used in this study are listed in Table S1.

### 2.2 Experimental animals

NLRP3‐KO mice were established by the Center for Comparative Medicine of the Institute of Experimental Animals of the Chinese Academy of Medical Sciences (Materials S1.7 for details). The 5XFAD mouse strain, Tg6799 (MMRRC Stock No:34840‐JAX), was donated by other laboratories and crossed with NLRP3‐KO mice to yield 5XFAD/NLRP3Het male mice. These mice were interbred to generate six genotypes: WT, NLRP3Het, NLRP3‐KO, 5XFAD, 5XFAD/NLRP3Het, and 5XFAD/NLRP3‐KO. The resulting offspring were genotyped by polymerase chain reaction (PCR) amplification of genomic DNA from tail biopsies and sequencing. The corresponding gene‐specific primer pairs and probe sequences are listed in Table S2. A total of 48 specific pathogen‐free (SPF) mice, including WT, 5XFAD, NLRP3‐KO, and 5XFAD/ NLRP3‐KO mice, were obtained. Each group consisted of six 2.5‐month‐old mice and six 9.5‐month‐old mice with the same proportion of females to males. All the animals were maintained in an SPF facility. Mice were housed and fed as previously described.^23^ All the animal studies were performed using protocols that were compliant with and approved by the Institutional Guidelines for the Care and Use of Laboratory Animals,^24^ Institute of Zoology (Beijing, China), and the Ethics Committee of the Laboratory of Animal Science of Peking Union Medical College (Approval No. QC19023). To reduce suffering before cervical dislocation, all animals were deeply anesthetized with pentobarbital sodium (50 mg/kg).

### 2.3 Cell lines and drug treatment

The murine microglial cell line BV2 (RRID: CVCL_0182) was purchased from Shanghai Mingjin Biotech Co., Ltd., and maintained in high‐glucose DMEM–pyruvate medium (11995065; Gibco) supplemented with 10% fetal bovine serum (10099141; Gibco) and 1% penicillin–streptomycin (15140122; Gibco). BV2 cells were serum‐starved, treated for 12 h, activated by incubation with 100 ng/mL LPS for 12 h to promote the transcription and translation of NLRP3 protein by activating the NF‐κB pathway, and then stimulated with 10 μM Aβ1‐42 oligomers alone for 4.5–24 h to generate an inflammatory response AD model in vitro.

### 2.4 Cell transfection and grouping

BV2 cells were transfected with lentivirus‐producing short‐hairpin RNAs targeting NLRP3 (NLRP3‐shRNAs) or p62 (p62‐shRNAs), HA‐NLRP3, Flag‐p62, or lentivirus control (Materials S1.8 for details). All the gene sequences were purchased from Viraltherapy Technologies. We infected BV2 cells with lentivirus for 36 h and screened them for specific antibiotics (Table S1). We obtained the following cell types: HA‐NLRP3, NLRP3‐shRNAs, Flag‐p62, p62‐shRNAs, HA‐NLRP3/Flag‐p62, and their respective controls.

### 2.5 Immunostaining

Details about this are provided in the materials and methods in Materials S1.1.

### 2.6 Flow cytometry

Cells were detached and stained with the violet eBioscience™ fixable viability dye eFluor™ 660 as a live–dead discrimination marker. Cells were stained with CD86 and CD16/32 as markers of M1 polarization status in microglia.^25^, ^26^, ^27^ Cells were diluted in eBioscience™ flow cytometry staining buffer and washed with flow cytometry staining buffer. Isotype controls were used for background staining. The cells were fixed with 4% paraformaldehyde and analyzed using a BD cytometer. Flow cytometry data were analyzed using FlowJo 10.0. A total of 10^5^ cells were collected from each group with three biological replicates.

### 2.7 Co‐IP and the western blots

Details of the materials and methods are provided in Materials S1.2. ImageJ was used to analyze the densitometry of protein bands in WB, and all the statistical graphs were based on quantitative experimental results.

### 2.8 Enzyme‐linked immunosorbent assay

The ELISA kit (Table S1) instructions were followed.

### 2.9 Mass spectrometry protein identification

Proteins were separated by SDS–PAGE on 4%–20% polyacrylamide gradient gels, stained with Coomassie brilliant blue, and the bands were cut out of the gel in a volume of 0.5–1 mm^3^. The MS analysis was performed by Novogene, China (see Materials S1.4, S1.9, and MS result.zip for details).

### 2.10 Quantitative real‐time PCR (qRT‐PCR)

Details of the materials and methods are provided in Materials S1.3. The primers used for qRT–PCR are listed in Table S3. The expression of each gene was calculated using the ΔΔCT method,^28^ and the genes of interest were normalized to the GAPDH reference gene.

### 2.11 RNA‐seq analysis

Total RNA was isolated from BV2 cells using TRIzol™ reagent (Novogene) and sequenced (see Materials S1.5, S1.9, and RNA‐seq analysis.zip for details).

### 2.12 Mouse behavioral tests

Mouse behavioral tests included Open‐Field Test (OFT).^29^, ^30^ Novel Object Recognition (NOR) test,^30^, ^31^ Morris Water Maze (MWM),^32^, ^33^, ^34^, ^35^ and Y maze.^23^ See Materials S1.6 for details. Data from the behavioral experiment were automatically recorded by a computer system Noldus EthoVision XT9 and completed by two researchers.

### 2.13 Statistical analysis

Data are expressed as the mean ± standard error of mean (SEM). Independent data (two groups) with normal distribution and homogeneity of variance were analyzed using Student's *t*‐test; otherwise, the non‐parametric test was analyzed using the Mann–Whitney test. Independent data with normal distribution were analyzed using one‐way followed by Tukey's post hoc test; otherwise the nonparametric test was analyzed using the Kruskal–Wallis test. Statistical analyses were performed using GraphPad Prism, version 8.0. Differences with *p* < 0.05 were considered significant. The following annotations were used to denote statistical significance levels: *p < 0.05, **p < 0.01, ***p < 0.001, ****p < 0.0001, #*p* < 0.05, ##*p* < 0.01, ###*p* < 0.001, ####*p* < 0.0001.

## 3 RESULTS

### 3.1 Restoration of cognitive function in 5XFAD/NLRP3‐KO mice

The results of the behavioral experiments showed that cognitive and learning functions were impaired in the 5XFAD group at 2.5 and 9.5 months, whereas cognitive and learning abilities were improved in the 5XFAD/NLRP3‐KO group.

**FIGURE 1:**
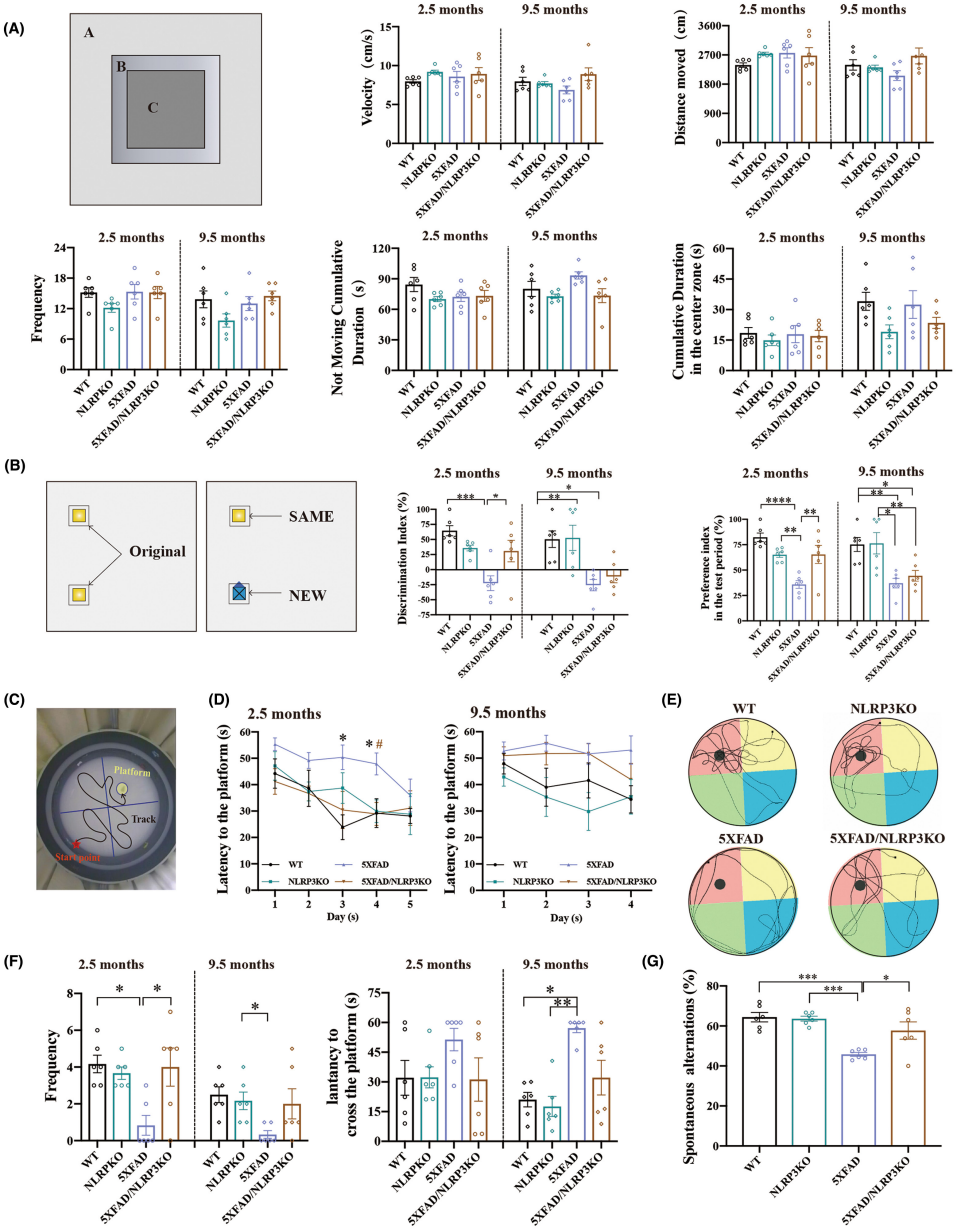
Restoration of cognitive function in 5XFAD/NLRP3‐KO mice. (A) Velocity, distance moved, frequency across the center zone (Zone C), not moving cumulative duration and cumulative duration in the center zone in the OFT. (B) Discrimination and preference indices during the test period in the NOR test. (C) The Morris water maze experimental facility. (D) Latency to the platform during the spatial training trials of MWM. (E) The swimming track in the probe trials of MWM. (F) Frequency across the platform and latency to cross the platform of MWM in the probe trials. (G) Spontaneous alternation in 9.5‐month‐old mice in the Y maze. *Represents statistical significance after comparing each group in D. # represents statistical significance after comparison between 5XFAD and 5XFAD/NLRP3KO group in D. All data are reported as mean ± SEM (*n* = 6, each group) for individual experiments. *p < 0.05, **p < 0.01, ***p < 0.001, ****p < 0.0001, #*p* < 0.05.

There was no significant difference in the OFT results between groups of the same age (Figure 1A). All the mice displayed normal spontaneous locomotor activity enabling subsequent completion of experiments to test their cognitive and learning abilities.

In the NOR test, the discrimination index and preference index during the test period were lower in the 5XFAD and 5XFAD/NLRP3‐KO groups, and there was a statistical difference between the WT and 5XFAD‐related groups. It is worth noting that the 5XFAD/NLRP3‐KO group had a higher average discrimination index and preference index in the test period than the 5XFAD group. There was a statistical difference between the 5XFAD and 5XFAD/NLRP3‐KO groups at 2.5 months (Figure 1B).

Figure 1C shows the equipment of the MWM. In the spatial training trials of the MWM, the latency to the platform of 5XFAD mice was longer than that of WT and 5XFAD/NLRP3‐KO mice. A significant difference was observed on the third and fourth day in 2.5‐month‐old mice (Figure 1D). In probe trials, the frequency to the platform for 5XFAD mice was lower than for the WT and 5XFAD/NLRP3‐KO groups and had significant difference at 2.5 months, while the latency to cross the platform of 5XFAD mice was higher than that of WT and 5XFAD/NLRP3‐KO groups in different age groups (Figure 1F). During the probe trials, as the experiment considered the physical ability of mice during swimming, the swimming time was limited to 60 s. If the mouse did not find the designated location within 60 s, it was also recorded as 60 s. Although there was no significant difference observed in the latency to cross the platform between the different age groups of 5XFAD and 5XFAD/NLRP3KO mice, at 9.5 months of age, it is worth noting that only one 5XFAD/NLRP3‐KO mouse was unable to reach the target location within 60 s, while four 5XFAD mice did not find the target location (right side of Figure 1F). This results indicate that a trend of cognitive function improvement is observed in the 5XFAD/NLRP3‐KO group compared to that in the 5XFAD group. Mice in the 5XFAD group showed the lowest ability to search for platforms. In contrast, the 5XFAD/NLRP3‐KO group showed a greater tendency to search for the platform purposefully, according to the tracking diagram depicting the platform‐searching activity of mice in the probe trials (Figure 1E).

We found that learning and memory ability was improved in mice; therefore, we verified the above experimental results in 9.5‐month‐old mice using the Y maze. The results revealed that 9.5‐month‐old mice with the 5XFAD genotype had a lower spontaneous change rate than age‐matched mice without the 5XFAD genotype. In addition, there was a significant difference between the 5XFAD, 5XFAD/NLRP3‐KO, and WT groups (Figure 1G). These results demonstrate that the 5XFAD/NLRP3‐KO group has improved their cognitive and learning abilities.

### 3.2 NLRP3 knockout reduces deposition of Aβ plaques in the AD models

**FIGURE 2:**
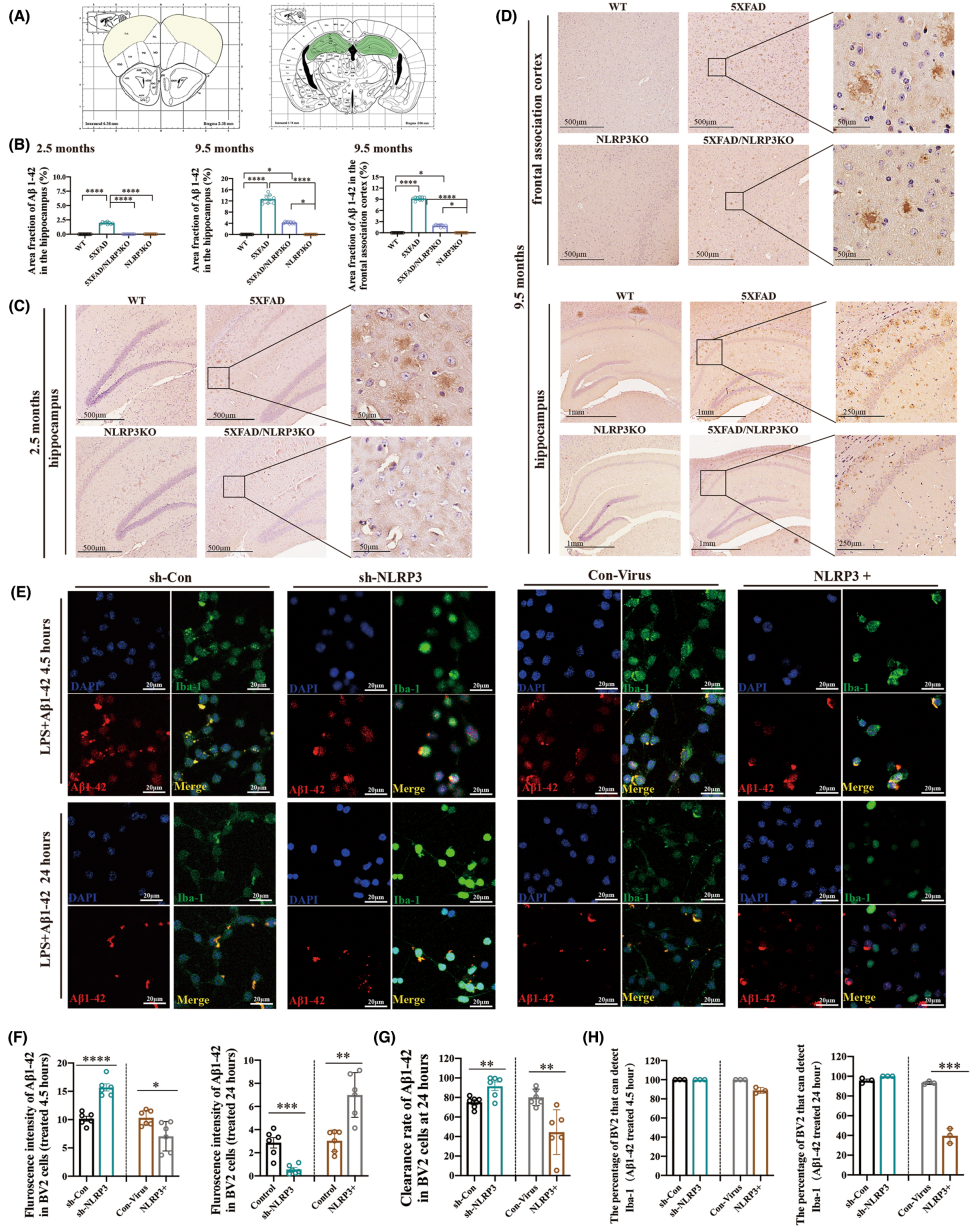
NLRP3 knockout reduces the deposition of Aβ plaques. (A) The location of the pathological section. (B) Area fraction of Aβ in the hippocampus and frontal association cortex. (C) Deposition of Aβ plaque in the hippocampus of 2.5‐month‐old mice. (D) Deposition of Aβ plaques in the hippocampus and frontal association cortex in 9.5‐month‐old mice. (E) Immunofluorescence results of BV2 cells under different treatment conditions. (F) Fluorescence intensity of Aβ1‐42 in BV2 cells treated with LPS (12 h) followed by Aβ1‐42 (4.5 h or 24 h). (G) The clearance rate of BV2 cells after treatment with LPS (12 h) followed by Aβ1‐42 (4.5 h or 24 h). (H) The percentage of BV2 cells which can detect Iba1 at different treatment conditions. Data are reported as the mean ± SEM. Three animals were selected randomly from each group, and three slices were selected from each animal (*n* = 9). 100 cells were selected in each group and repeated the experiment 3 times (*n* = 3). *p < 0.05, **p < 0.01, ***p < 0.001, ****p < 0.0001.

Deposited Aβ plaques are characteristic pathological changes in AD that induce neuroinflammation and are neurotoxic. Since the hippocampus and frontal cortex are most closely related to learning and memory, we mainly analyzed these brain regions. The frontal association cortex was located in the coronal plane 3.08–2.58 mm from bregma. The hippocampus was located in the coronal plane −1.82 to −2.54 mm from bregma (Figure 2A). Immunohistochemistry results showed that Aβ plaques were deposited in the hippocampus of 5XFAD mice at 2.5 months. There was a statistically significant reduction of Aβ plaque deposition in hippocampus of 5XFAD/NLRP3‐KO mice than that of 5XFAD mice (Figure 2B,C). No Aβ plaques were found in the frontal cortex of 2.5‐month‐old mice (data not shown). The distribution area of deposited Aβ plaques in the hippocampus and frontal cortex of 5XFAD mice at 9.5 months was the highest. There was a statistically significant reduction in hippocampas and frontal cortex Aβ plaque deposition in 5XFAD/NLRP3‐KO mice compared to 5XFAD mice at 9.5 months (Figure 2B,D). The mean diameter of Aβ plaques in 5XFAD was significantly larger than in the 5XFAD/NLRP3‐KO group in Figure 2D (Figure S1j). This demonstrated that NLRP3 knockout reduces the deposition of Aβ plaques in in vivo AD models.

Then, we treated BV2 cells with LPS following the Aβ1‐42 oligomer to verify the ability of microglia to phagocytose Aβ1‐42 and cellular clearance function in vitro. The whole‐cell contour was marked using Iba‐1 (a microglia‐specific cell membrane protein). However, BV2 cells in pyroptosis can be labeled with DAPI because their cell membranes burst and their cell contents spilling out.

Immunofluorescence results of BV2 cells in different groups under different treatment conditions (Figure 2E). The fluorescence intensity of Aβ1‐42‐treated BV2 cells at 4.5 h represented the phagocytic capacity of Aβ1‐42, and the higher the fluorescence intensity, the stronger the phagocytic capacity. The fluorescence intensity of Aβ1‐42‐treated BV2 cells at 24 h represents the degree of degradation of Aβ1‐42 in BV2 cells.

The results showed that the phagocytic function of Aβ1‐42 was enhanced in the sh‐NLRP3 cell group, whereas the phagocytic function of Aβ1‐42 was decreased in the NLRP3 overexpression cell group (Figure 2F). The ability of BV2 cells to clear Aβ1‐42 declined in the NLRP3 overexpression group (Figure 2G). In addition, the number of cells with detectable Iba1 fluorescence signal decreased in the NLRP3 overexpression group, indicating that pyroptosis occurred (Figure 2H). Therefore, reduction of NLRP3 expression inhibits pyroptosis induced by Aβ1‐42 stimulation and maintains phagocytosis of BV2 cells.

### 3.3 Expression of NLRP3 promotes pro‐inflammatory status and pyroptosis of microglia

As NLRP3 is the rate‐limiting protein for the formation of the NLRP3 inflammasome, the pro‐inflammatory factors released by inflammasomes affect the microglial M1 polarization state. The results showed that activation of the NLRP3 inflammasome promotes the pro‐inflammatory status and pyroptosis of microglia in vivo and in vitro.

In our study, NLRP3 inflammasome activation was induced in the 5XFAD mouse model and BV2 cells by treatment with LPS and Aβ1‐42 oligomers. Changes in NLRP3 expression, caspase‐1 cleavage, and IL‐1β cleavage were quantified by WB or ELISA (Figure S1).

**FIGURE 3:**
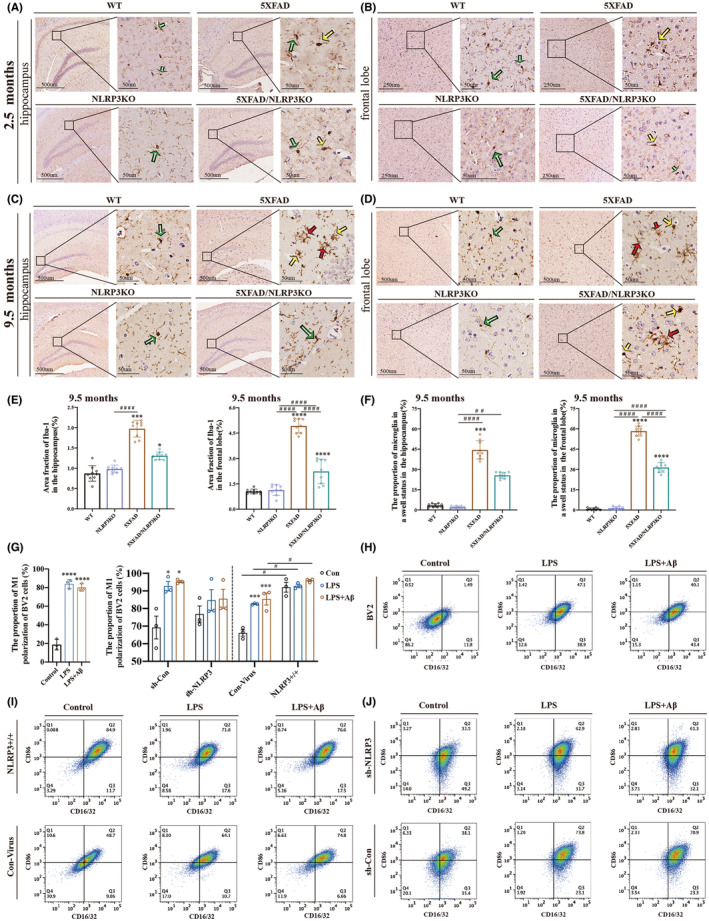
NLRP3 knockout reduces microglia pyroptosis. Microglia with different colored arrows in A–D represent different functional states. Microglia indicated by the green arrow have small soma and elongated synapses that are in the resting state. Microglia pointed by the yellow arrow, with an enlarged soma and short and thick synapses, are in an activated state. Microglia indicated by the red arrow are swollen with no clear nuclei, indicating pyroptosis. (A, B) Morphology of microglia in the hippocampus and frontal association cortex of 2.5‐month‐old mice. (C, D) Morphology of microglia in the hippocampus and frontal association cortex of 9.5‐month‐old mice. (E) Area fraction of Iba‐1 in C and D. (F) The proportion of microglia in a swollen state in C and D. (G–J) Flow cytometry was used to identify the pro‐inflammatory state of BV2 cells with different levels of NLRP3 expression under different drug treatments. Data are reported as the mean ± SEM. Three animals were selected randomly from each group, and three slices were selected from each animal (*n* = 9).100 microglia were collected from each slice. A total 10^5^ cells were selected in each group and repeated the experiment 3 times of individual experiments in vitro. *p < 0.05, ***p < 0.001, ****p < 0.0001, #*p* < 0.05, ##*p* < 0.01, ####*p* < 0.0001. *Was used to indicate statistical significance when comparing within the group to the control (WT), while a # was used to indicate statistical significance when comparing between groups.

The pathological results in Figure 3 show that microglia had different morphologies under different pathophysiological conditions, and the morphological differences indicate that microglia have different physiological functions.

We observed only microglia in the resting and activated states in the hippocampus (Figure 3A) or frontal association cortex (Figure 3B) in 2.5‐month‐old‐old mice. In comparison, pyroptosis was observed in the hippocampus and frontal regions of the 9.5‐month‐old mice (Figure 3C,D). The area fraction of microglia in the hippocampus and frontal association cortex was significantly higher in 5XFAD mice than that in 5XFAD/NLRP3‐KO mice and WT mice at 9.5 months (Figure 3E). The proportion of microglia in a swollen state was significantly increased in both the hippocampus and frontal cortex of the 5XFAD mice group compared with the WT and 5XFAD/NLRP3‐KO mice at 9.5 months (Figure 3F). In the in vitro experiments, flow cytometry of BV2 cells in the AD inflammation model showed polarization to a pro‐inflammatory status (Figure 3G,H). The pro‐inflammatory status of BV2 cells was significantly increased in the NLRP3 overexpression group (Figure 3G,I). When NLRP3 was overexpressed, the cells were transformed to a pro‐inflammatory state, and the number of pro‐inflammatory functional BV2 cells tended to increase under the stimulation of LPS and Aβ1‐42 oligomers.

Although no significant difference was found between the sh‐NLRP3 group and the control group, the sh‐NLRP3 group had a lower mean percentage of BV2 cells in the pro‐inflammatory state than the control group. In additon, there was no statistical difference within the sh‐NLRP3 group, which was different from that in the sh‐Con group, indicating that the reduction of NLRP3 expression has a certain inhibitory effect on the pro‐inflammatory function of BV2 cells (Figure 3G,J). The results showed that the expression level of NLRP3 was the main factor affecting the pro‐inflammatory polarization of microglia.

### 3.4 Co‐IP of NLRP3 complex from in vitro AD models analyzed by mass spectrometry

**FIGURE 4:**
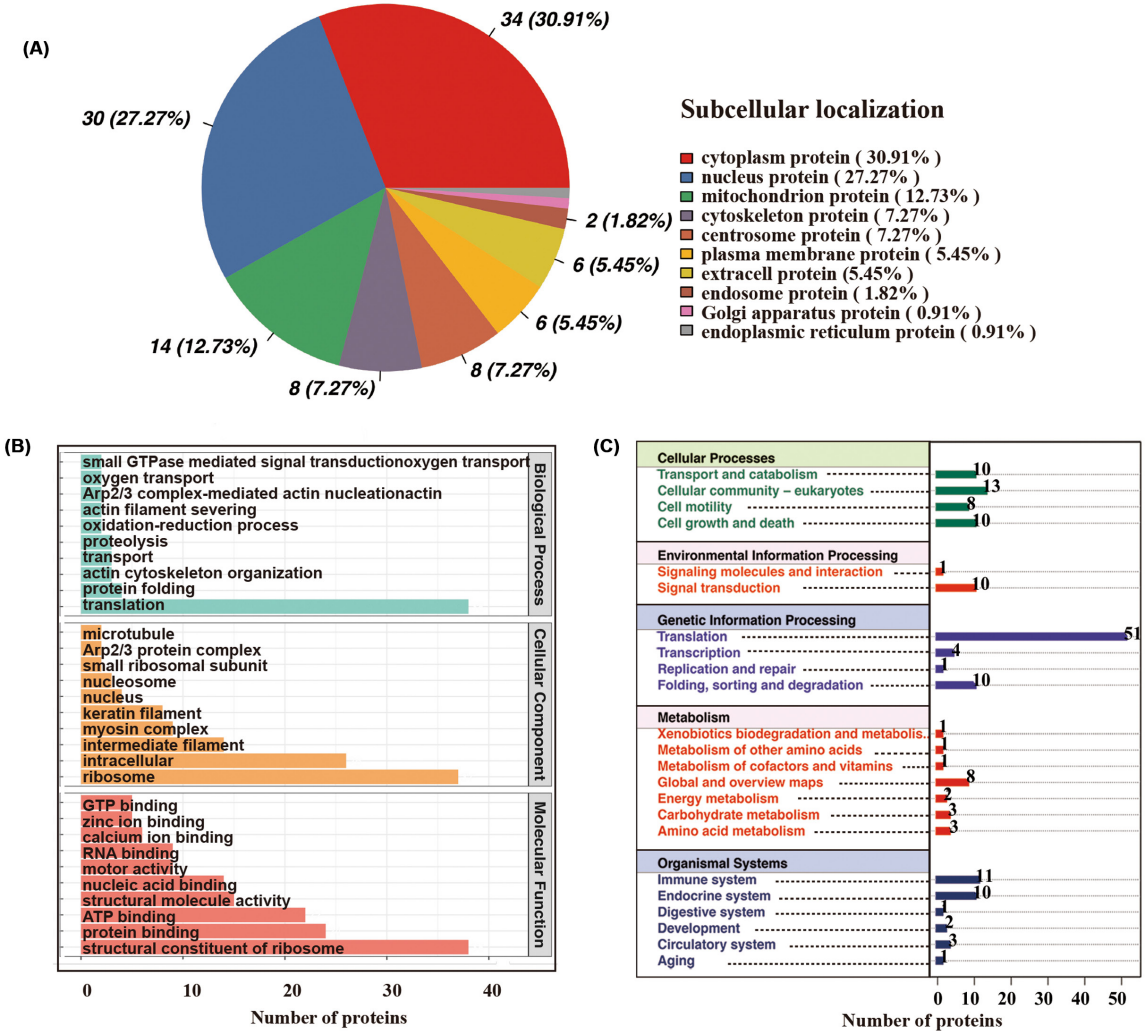
Co‐IP of NLRP3 complex in BV2 cells of an AD inflammation model and mass spectrometry protein analysis. (A) Localization of the NLRP3 co‐IP complex in the subcellular in the LPS + Aβ1‐42 treated BV2 group. (B) GO enrichment analysis of the proteins in A. (C) The KEGG pathway annotation of proteins in A. The resulting spectra from each fraction were searched separately against the Mus_musculus_uniprot_2019.01.18. fasta (85,188 sequences) database using the following search engine: Proteome Discoverer 2.2 (PD 2.2, Thermo).

This study confirmed that the expression level of NLRP3 affects the polarization state of microglia and plays a significant role in the progression of AD. Next, we used co‐IP combined with mass spectrometry to explore proteins involved in regulating NLRP3. Over 100 identified co‐IP binding proteins (specifically bound to NLRP3 and nonspecifically bound to the antibody) were matched in the database with peptides analyzed by mass spectrometry to complete protein identification. NLRP is mainly distributed in the cytoplasm. In this study, the NLRP3 protein complex bound by co‐IP was mainly distributed in the cytoplasm which is consistent with the subcellular localization of NLRP3 (Figure 4A). Except for functions related to ribosomal translation, we found protein folding for biological process (BP), intracellular proteins for cellular components (CC), and protein‐binding for molecular functions (MF) at the head of the list (Figure 4B) in GO enrichment analysis. In addition, we found interacting proteins related to the immune system, protein folding, protein sorting and degradation, and cellular processes in KEGG pathway annotation analysis (Figure 4C). This indicates that NLRP3 complex‐related proteins are involved in a variety of biological functions and signaling pathways.

We further screened for proteins that bind to NLRP3‐specific antibodies by comparing IgG control and LPS‐treated groups, Among the remaining proteins with bioregulatory functions, we selected p62 as the target protein (Table 1; see Materials S1.4, S1.9 and MS result.zip for details).

**TABLE 1:** Proteins that were screened and their respective functions.

Protein	Gene	Relative expression	Description
Q5PR73	Diras2	154219776.00	GTP‐binding protein Di‐Ras2
Q8R4B8	Nlrp3	106144595.30	NACHT, LRR and PYD domains‐containing protein 3
P20029	Hspa5	15856161.75	Endoplasmic reticulum chaperone BiP
P57722	Pcbp3	6824189.50	Poly(rC)‐binding protein 3
Q9JJ28	Flii	6678268.09	Protein flightless‐1 homolog
E9PYL9	Gm10036	6265178.00	Predicted gene 10,036
G4V4Z1	gag‐pro‐pol	6176814.63	Gag‐pro‐pol polyprotein
Q61782	X	5278309.00	Type I epidermal keratin mRNA, 3end (Fragment)
Q3TIP8	Clic1	4288602.50	Chloride intracellular channel protein
Q8C2Q7	Hnrnph1	4023988.75	Heterogeneous nuclear ribonucleoprotein H
Q8C788	Snx18	3593210.25	Sorting nexin
E9Q8L9	Rab11fip1	3448920.75	Rab11 family‐interacting protein 1
P19157	Gstp1	3440789.75	Glutathione S‐transferase P 1
Q64337	Sqstm1/P62	2732858.03	Sequestosome‐1
B0LAC7	Myl6	2665806.50	Myosin light polypeptide 6 alkali smooth muscle and non‐muscle protein (Fragment)
Q921V3	Nfs1	2330083.75	Nitrogen fixation gene 1 (*S. cerevisiae*)
Q7TMK9	Syncrip	2134397.50	Heterogeneous nuclear ribonucleoprotein Q
Q5M9J8	Rpl28	2116607.50	MCG13936
Q5SF07	Igf2bp2	1372867.13	Insulin‐like growth factor 2 mRNA‐binding protein 2
Q8BFZ3	Actbl2	1366048.38	Beta‐Actin‐like protein 2
A2AWP8	Arhgef10l	1284277.50	Rho guanine nucleotide exchange factor 10‐like protein
P62960	Ybx1	772352.75	Nuclease‐sensitive element‐binding protein 1

### 3.5 P62 interacts with NLRP3 and regulates the polarization status of microglia

**FIGURE 5:**
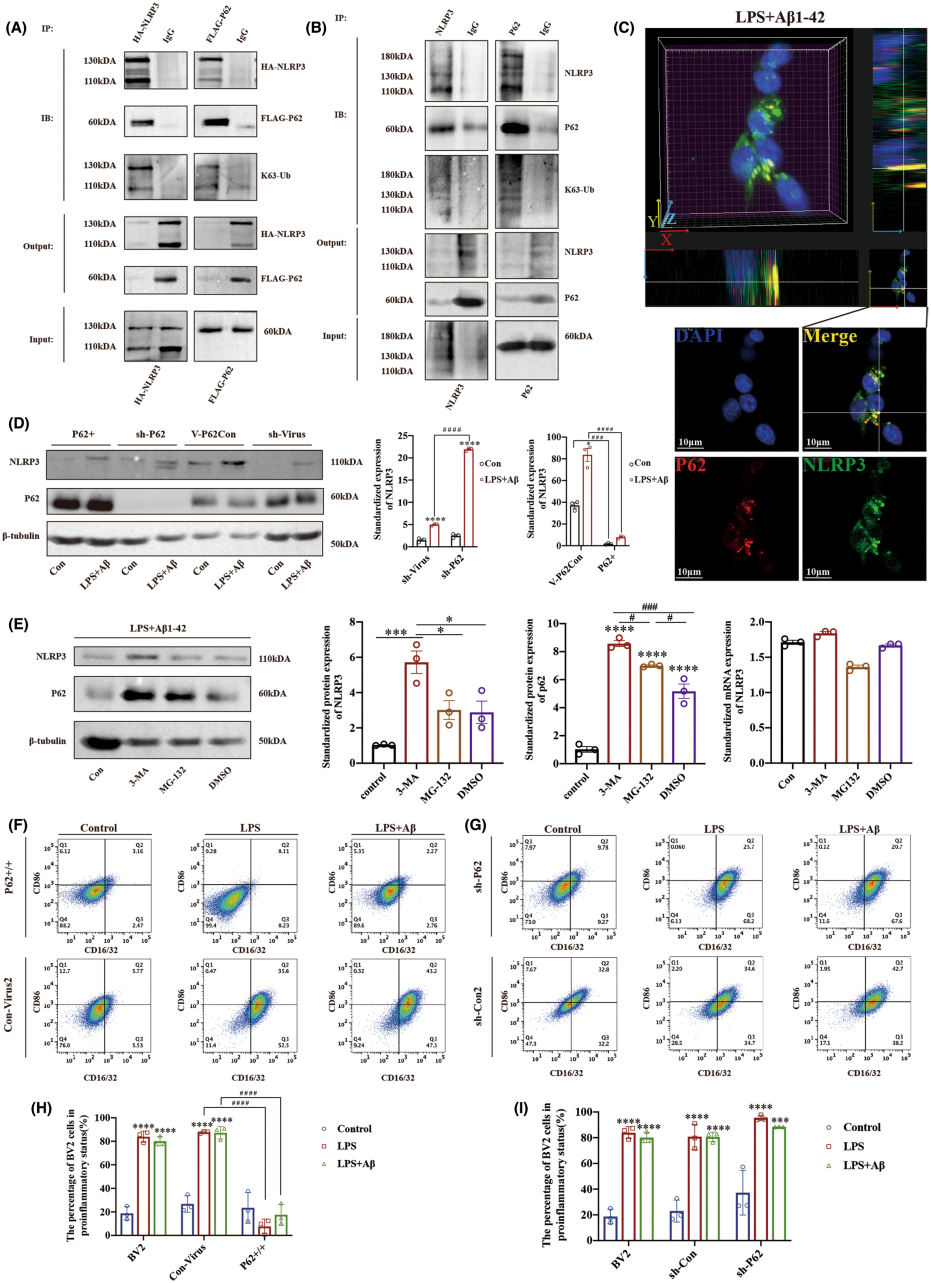
P62 interacts with NLRP3 and regulates the polarization state of microglia. (A, B) Co‐immunoprecipitation was performed using exogenous and endogenous NLRP3 and p62. BV2 cells were infected with lentivirus over‐expressing HA‐NLRP and FLAG‐p62 and treated with LPS followed by Aβ1‐42 oligomers. (C) IF co‐localization of NLRP3 and p62 in LPS‐and Aβ1‐42‐treated BV2 cells. (D) WB was performed to detect the protein level of NLRP3 in BV2 cells with different levels of p62 expression. (E) Protein and mRNA expression levels in BV2 cells treated with 3MA, MG132, or DMSO, followed by treatment with LPS and Aβ1‐42 oligomers. (F, G) Flow cytometry was used to identify the pro‐inflammatory state of BV2 cells with different levels of p62 expression under different drug treatments. (H, I) The proportion of BV2 cells in a pro‐inflammatory status in Figure 3F,G. 10^5^ Cells were selected in each group in Figure 3F,G. Data are reported as the mean ± SEM (*n* = 3) of individual experiments. * Was used to indicate statistical significance when comparing within the group to the control, while a # was used to indicate statistical significance when comparing between groups.*p < 0.05, ***p < 0.001, ****p < 0.0001, #*p* < 0.05, ####*p* < 0.0001.

We confirmed that p62 interacts with NLRP3 by exogenous and endogenous co‐IP and IF co‐localization experiments. We transfected BV2 cells with HA–NLRP3 and FLAG‐p62 lentiviruses to overexpress exogenous target proteins. BV2 cells were treated with LPS and Aβ1‐42 oligomers to induce endogenous and exogenous NLRP3 expression, respectively. The results showed an interaction between HA–NLRP and FLAG‐p62 proteins (Figure 5A) and the NLRP3 and p62 (Figure 5B). In addition, K63‐ubiquitinated NLRP3 was present in the protein complexes (Figure 5A,B). The protein interaction relationship was also confirmed by IF colocalization experiments in LPS‐ and Aβ1‐42 oligomer‐treated BV2 cells (Figure 5C). We further verify the regulatory relationship between NLRP3 and p62 by regulating the expression of p62 by lentivirus transfection. Transfection with a lentivirus carrying an empty vector served as a control. The result showed that p62 negatively regulated NLRP3 protein expression (Figure 5D).

We further explored whether p62 participates in NLRP3 protein degradation through the ALP. We treated BV2 cells with 3‐MA,^36^ dissolved in DMSO as an ALP inhibitor, with the DMSO vehicle as a control. Meanwhile, MG‐132, as an inhibitor of the ubiquitin‐proteasome system (UPS) for protein degradation, was used as the control.

The mRNA level of NLRP3 was not significantly different from that in the 3‐MA‐, MG‐132‐, and DMSO‐treated groups, whereas the protein level of NLRP3 was significantly higher in 3‐MA‐treated BV2 cells than in MG‐132‐ or DMSO‐treated BV2 cells. Therefore, we concluded that NLRP3 was degraded by ALP (Figure 5E).

Next, we analyzed the effect of p62 on microglial polarization using flow cytometry. The number of CD16/32 or CD86 cells selected for the statistical sample of proinflammatory cells and data^25^, ^26^, ^27^ and the nontransfected BV2 cell sample were the same as shown in Figure 3G. The results showed that the pro‐inflammatory status of BV2 cells decreased after p62 overexpression (Figure 5F,H). As a supplement to this part of the experiment, we downregulated p62 in BV2 cells. Both the sh‐Con and sh‐P62 groups exhibited elevated levels of pro‐inflammatory status BV2 cells in response to both LPS and LPS + Aβ stimulation, as compared with their respective control groups. Although there was no statistically significant difference between the sh‐p62 group and the sh‐con group, we found the mean value (LPS: 95.34%, LPS + Aβ: 88.43%) of the pro‐inflammatory status of cells in the sh‐p62 group is higher than the sh‐con group (LPS: 80.77%, LPS + Aβ: 80.63%) (Figure 5G,I).

### 3.6 Results of RNA‐seq analysis supported the activation of p62‐related autophagy pathways in the AD inflammation in vitro model

**FIGURE 6:**
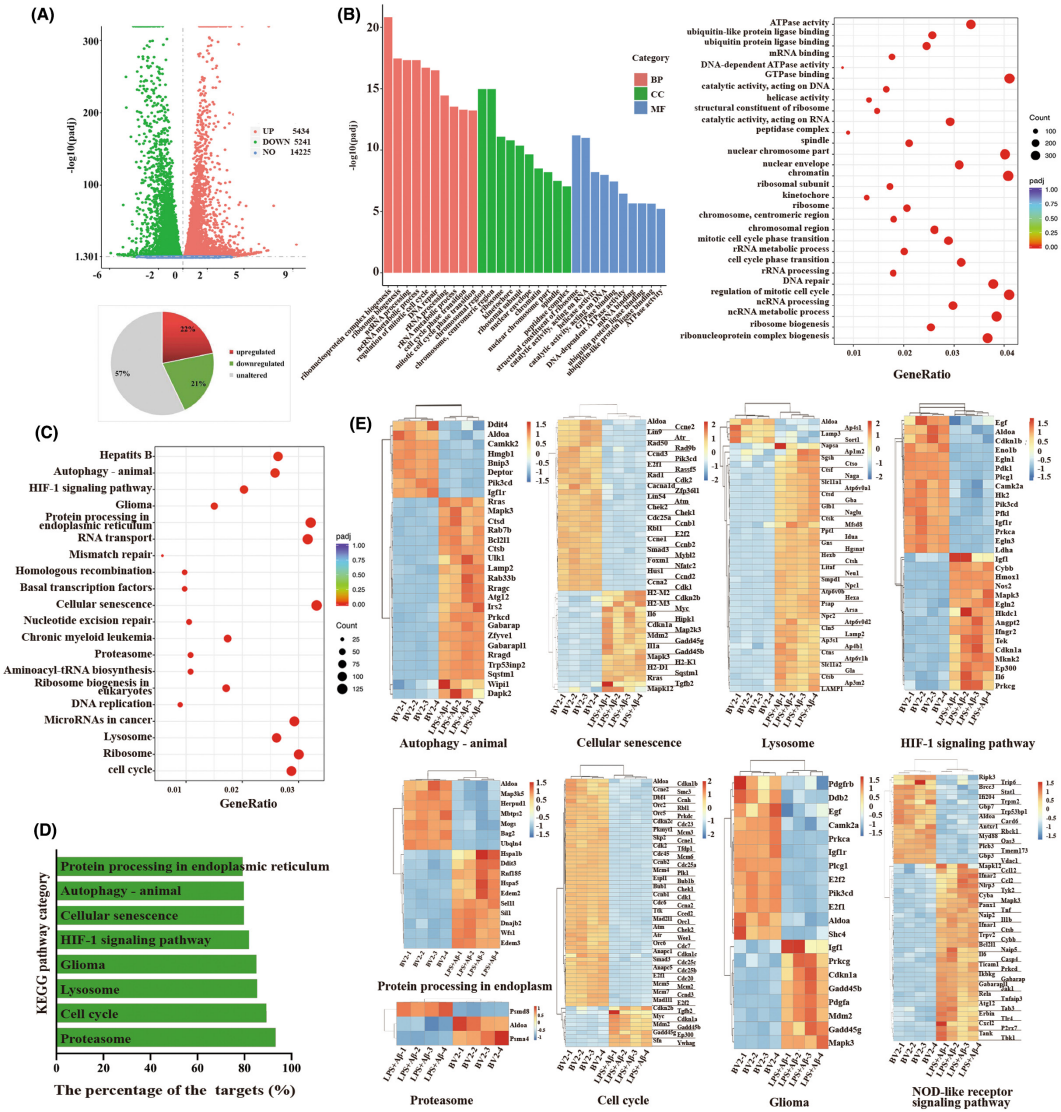
RNA‐seq results supported the activation of p62‐related autophagy pathways in the AD inflammation in vitro model. (A) Differential gene expression analysis in BV2 cells treated with LPS followed by Aβ1‐42 and control groups. (B) The GO enrichment analysis of the differentially expressed genes is shown in A (the most significant 30 terms were selected to draw a scatter diagram for display, *p*_adj_ < 0.05). BP: biological processes, CC: cellular components, MF: molecular functions. (C) The KEGG pathway analysis of differentially expressed genes (the most significant 20 terms were selected to draw a scatter diagram for display, *p*_adj_ < 0.05) in A. (D) The percentage of target genes of the eight selected pathways. (E) Heatmaps of differentially expressed genes in the signaling pathways of D and the NOD‐like receptor signaling pathway(|logFC| > 1.5, *p*_adj_(*q*) < 0.01).

To further investigate the effect of changes in gene expression in the in vitro inflammatory model of AD, RNA‐seq analysis was performed. The results showed that among the differentially expressed genes (DEGs), 5434 were upregulated, and 5241 were downregulated (Figure 6A). In the GO enrichment analysis, we listed the top 10 functions for BP, intracellular proteins for CC, and protein binding for MF (Figure 6B). Twenty statistically different functional pathways were listed according to the KEGG analysis (Figure 6C).

**FIGURE 7:**
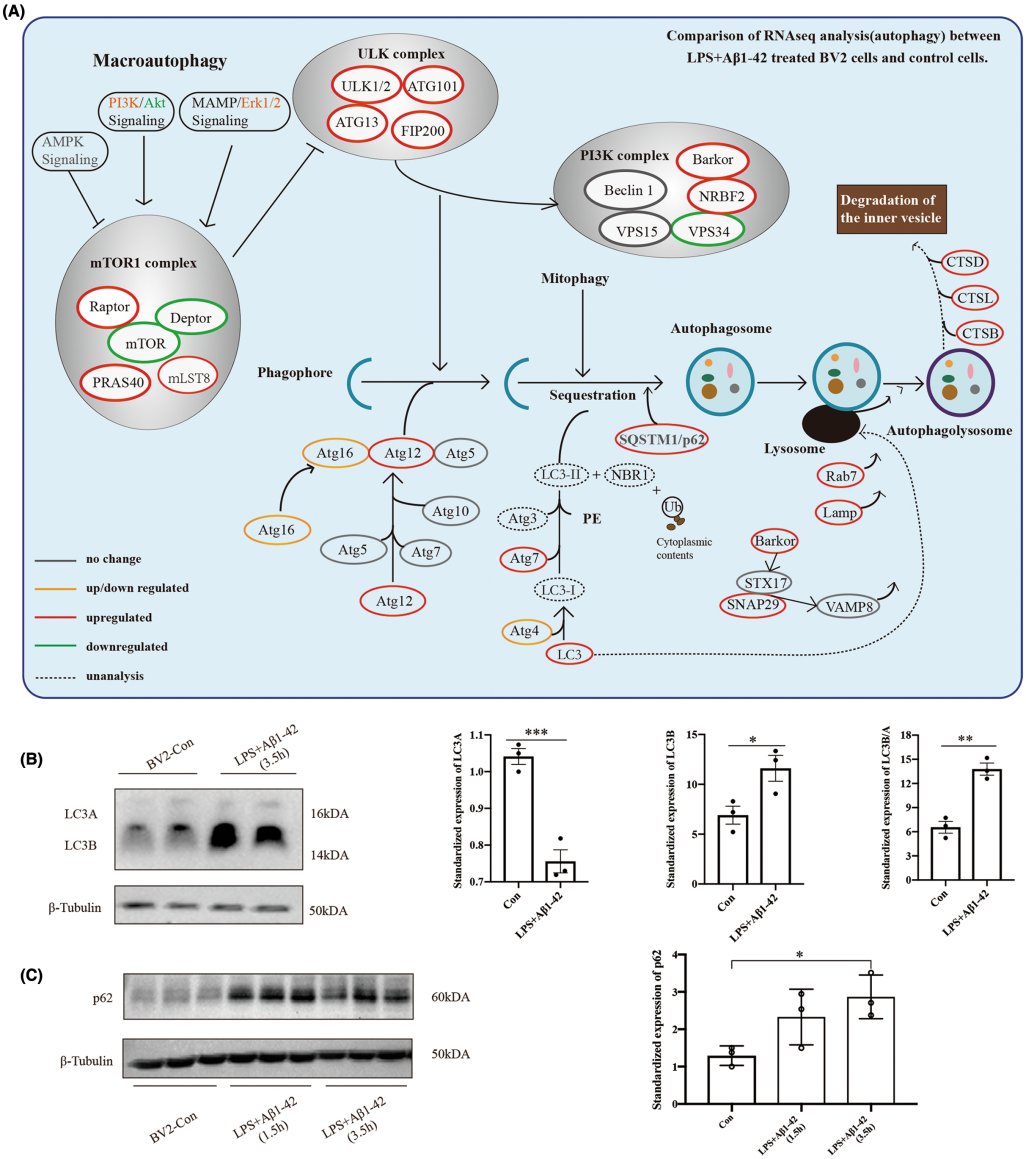
The autophagy pathway was activated in the in vitro AD models. (A) The mRNA expression levels of autophagy pathway‐related molecules according to RNA‐seq results in Figure 6E. (B, C) Protein levels related to the autophagy pathway in the AD model in vitro. Data are reported as the mean ± SEM (*n* = 3) of individual experiments. *p < 0.05, **p < 0.01, ***p < 0.001.

We focused on the eight KEGG‐defined pathways associated with cell damage, including autophagy (animal), cellular senescence, lysosomes, protein processing in the endoplasm, the cell cycle, and glioma. We calculated the percentage of target genes (Figure 6D) and depicted them on a heatmap for each term. The NOD‐like receptor signaling pathway was also included (Figure 6E, |logFC| > 1.5, padj(*q*) < 0.01). The RNA‐seq analysis results for BV2 treated with the LPS only and the LPS plus Aβ1‐42 are shown in Figure S2. Changes in the NF–κB pathway and the AD‐related pathway (Figure S2D) were found in both groups, demonstrating that LPS + Aβ drug stimulation of BV2 cells is a reliable model of AD inflammation. In addition, we list the down/upregulated expression of upstream and downstream genes related to p62 in the autophagy pathway (Figure 7A).

### 3.7 The autophagy pathway was activated in vitro AD models

According to the results of RNA seq in Figure 6E, we showed the specific mRNA expression of autophagy‐related pathways in AD models in vitro, with different colors representing changes in expression levels compared to the control group. The results showed that mRNA expression of LC3, Atg3/7/12, and LAMP in the in vitro AD model increased and Akt–mTOR‐related mRNA changed, which indicated Akt–mTOR pathway participated in the process of autophagy (Figure 7A). In addition, the expression change of LC3A, LC3B, LC3B/A, and p62 indicated that the autophagy pathway was activated in the in vitro AD model (Figure 7B,C).

## 4 DISCUSSION

Deposited Aβ plaques that cause neurotoxicity can activate the NLRP3 inflammasome^37^ and induce an inflammatory response of microglia in the central nervous system; therefore, it is important to maintain the phagocytosis of microglia and reduce their pro‐inflammatory state. In this study, we found that reducing NLRP3 expression improved cognitive dysfunction in the early and middle stages of 5XFAD mice. In addition, we verified that p62 could regulate the assembly and synthesis of the NLRP3 inflammasome through various biological methods and explored its regulatory mechanism.

In our study, 5XFAD/NLRP3KO mice were used because the Aβ plaque deposition time was earlier in the 5XFAD mouse strain than in the APP/PS1 mouse strain. We observed behavioral and pathological changes in brain tissue in early and middle‐stage mice, and the results were consistent with the conclusion of the Heneka's study,^5^ involving APP/PS1/NLRP3‐KO mice. This provides a theoretical foundation for inhibiting the assembly and synthesis of NLRP3 inflammasome as a strategy to improve AD.

Microglia recognize and phagocytose deposited Aβ plaques to alleviate neuronal degeneration and necrosis caused by neurotoxicity. However, bidirectional regulation of microglia by Aβ plaques plays an essential role in regulating the functional status homeostasis of microglia and affects the progression of AD. Aβ plaques are Nod‐like receptor activators that induce the assembly and activation of the NLRP3 inflammasome in microglia. Thus, microglia convert to a pro‐inflammatory state, promoting persistent and excessive inflammatory responses, and leading to an immune response imbalance, further aggravating neuronal damage. Exploring the molecular mechanism that regulates the assembly and synthesis of NLRP3 inflammasome is critical.

To identify molecules that participate in the formation of the NLRP3 inflammasome, we used Co‐IP/mass spectrometry to search for molecular proteins that could interact with NLRP3. The proteins in the mass spectrometry results were mainly expressed in the cytoplasm and participated in transcription and protein‐binding BP. The KEGG signaling pathway contained activating signaling pathways related to the immune system and metabolism. Based on these results, we selected 22 proteins most likely to interact with NLRP3. One of these 22 proteins, p62, is an autophagy‐related protein involved in protein degradation. Previous studies^19^, ^38^ have shown that p62 recognizes K63‐ubiquitinated proteins and degrades them via the autophagosome pathway. Shi et al (2012) found that p62 binds to part of the inflammasome in human acute monocytic leukemia cells (THP).^39^ We confirmed the interaction between NLRP3 and p62 using endogenous and exogenous co‐IP, colocalization, and immunofluorescence in BV2 cells. We also verified the binding of K63‐ubiquitin to NLRP3 protein in the protein complex using a co‐IP assay. To confirm that p62 participates in the degradation of NLRP3, we regulated the expression of p62 in BV2 cells by transfection with lentiviral plasmid vectors and detected the NLRP3 protein levels by WB. The results showed that p62 negatively regulated the expression of NLRP3.

The two major pathways that may contribute to the degradation of NLRP3 are the UPS and the ALP.^21^ To test whether ALP contributes to NLRP3 degradation, we used 3MA as an ALP inhibitor and measured NLRP3 mRNA and protein levels.^40^ In addition, we used MG‐132 as a control group to investigate whether the proteasome pathway is involved in the degradation of NLRP3.^41^ The results showed that the NLRP3 mRNA expression levels were not significantly different between the two protein degradation pathways. However, the NLRP3 protein level in the 3‐MA‐treated group was higher than that in other groups, suggesting that the degradation of NLRP3 was mainly through ALP in microglia activated by LPS + Aβ1‐42. The polarization status of BV2 cells was determined after up/downregulation of the expression of p62. The results showed that p62‐regulated NLRP3 protein levels and the generation of the NLRP3 inflammasome, which affected the polarization state of microglial cells. Targeting p62 can maintain the phagocytic ability of microglia and retard the pathological processes of AD.

P62 participated in the degradation of NLRP3 through ALP was verified in this study. We further explore and analyze the signaling pathways that might be involved in the AD inflammation model using transcriptome analysis. This part of the results testified to the above research content and provided ideas for future research. The results showed that in addition to the autophagy pathway, the HIF‐1 pathway, cellular senescence, lysosomes, protein processing in the endoplasm, cell cycle, and glioma are also involved in the regulation of the AD inflammatory model, which can provide a theoretical basis for exploring the mechanisms of AD inflammation in the future. The biological process includes activating a variety of signaling pathways, thus providing a new range of opportunities for inhibiting the inflammatory response. Meanwhile, we focused on molecular changes associated with the autophagy pathway, such as LC3A, LC3B, LC3B/A, and p62. As p62 is involved in the ALP, we aim to further investigate which molecules mediate the autophagy process in microglia cells in AD. This can provide some ideas and directions for future research. Through the KEGG analysis of differentially expressed genes in RNA‐seq analysis, we found that the autophagy pathway exists in BV2 cells after LPS + Aβ stimulation (Figure 6D). Analysis of differentially expressed genes in the autophagy pathway revealed differential changes in genes in the Akt–mTOR pathway (Figures 6E and 7), which meet the conditions for autophagy initiation. This indicates that the Akt–mTOR pathway was activated in the microglia and induced p62 participation in NLRP3 protein degradation.

In this study, we found that p62‐mediated autophagy degradation of the NLRP3 protein can delay the progression of AD by reducing the inflammatory response, providing a theoretical basis for the targeted treatment of AD. Studies have shown that AD can be diagnosed early using noninvasive urine exosome detection.^42^ Combined with our study, reducing the early inflammatory response in AD can slow the process of AD. Therefore, it is necessary to identify a more effective treatment for this inflammatory response. In future studies, new drugs can be explored to treat AD through NLRP3 inhibitors or p62 agonists to reduce the expression of the NLRP3 protein to reduce the inflammatory response and delay the progression of AD.

## AUTHOR CONTRIBUTIONS

All the authors contributed to the study's conception and design. Professor Chuan Qin and Ling Zhang were responsible for the overall design and resulted in an analysis of the project. Material preparation, data collection, and analysis were performed by Dongyuan Zhang, Yu Zhang, Jirong Pan, and Jingjing Cao. The first draft of the manuscript was written by Dongyuan Zhang, Yu Zhang, Writing – review, and editing Yu Zhang, Xiuping Sun, Xianglei Li. Funding acquisition, Chuan Qin, Ling Zhang, Yu Zhang. All the authors commented on previous versions of the manuscript. All the authors read and approved the final manuscript.

## FUNDING INFORMATION

CAMS Innovation Fund for Medical Science, Grant Number: 2021‐I2M‐1‐034. National Natural Science Funding of China: 31970510, 81941012, 82161138027. National Natural Science Foundation Of China/National Research Foundation Collaboration Call For Project Proposals. Young Elite Scientist Sponsorship Program by CAST (2019QNRC001).

## CONFLICT OF INTEREST STATEMENT

The authors have no relevant financial or nonfinancial interests to disclose.

## CONSENT FOR PUBLICATION

The study did not involve human subjects, so there is no consent to participate.

## Supporting information


Figure S1

Figure S2

Data S1

Data S2

Data S3

## Data Availability

The datasets generated during and/or analyzed during the current study are available from the corresponding author upon reasonable request.
