# Supplementary figures and images for: Degradation of NLRP3 by p62‐dependent‐autophagy improves cognitive function in Alzheimer's disease by maintaining the phagocytic function of microglia
Source: CNS Neurosci Ther. 2023 Apr 18;29(10):2826–42. doi: 10.1111/cns.14219 (PMC10493665; doi:10.1111/cns.14219)

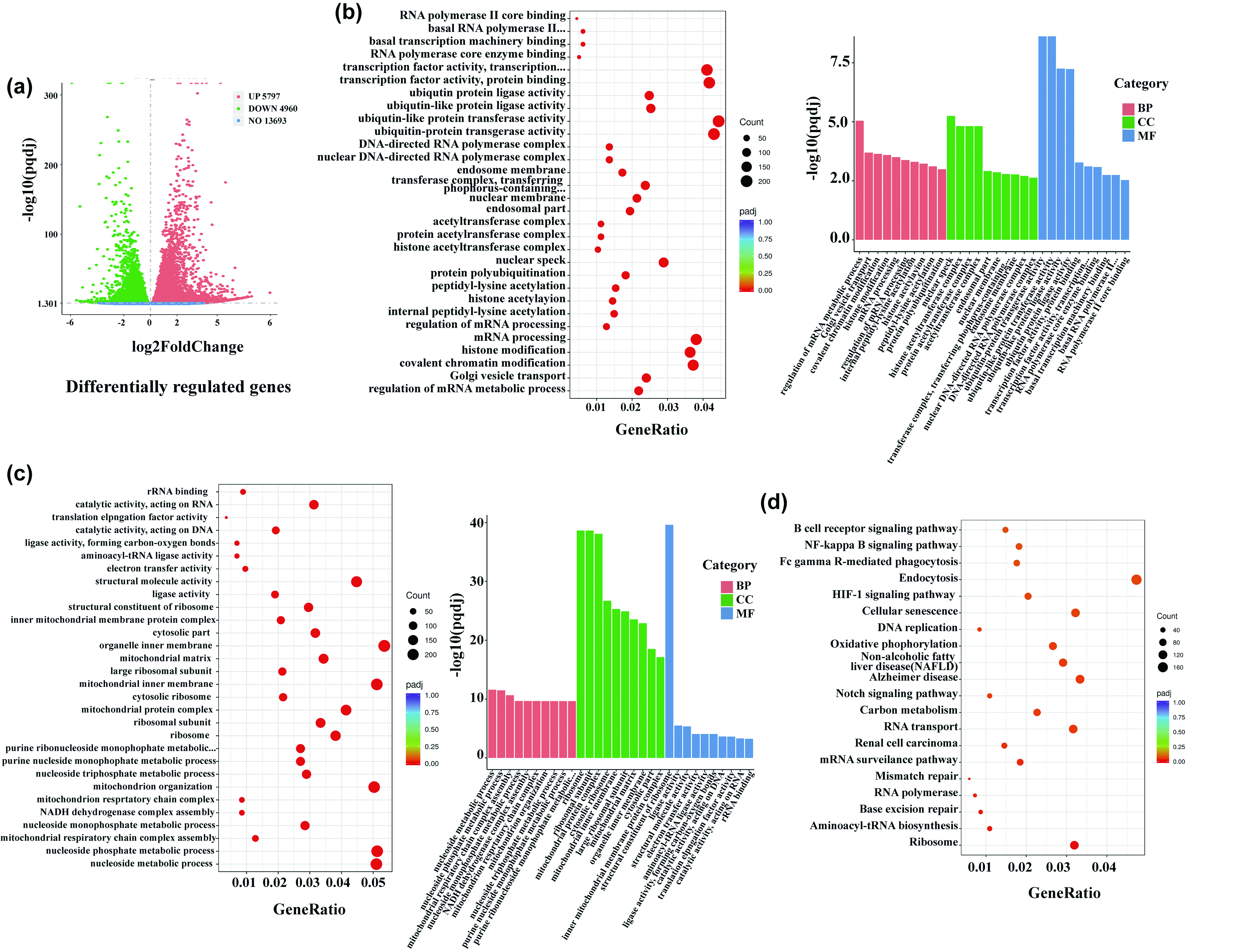

Supplement: Supplementary file 2 — Figure S2 [file CNS-29-2826-s003.tif]
