# Supplementary material for: Degradation of NLRP3 by p62‐dependent‐autophagy improves cognitive function in Alzheimer's disease by maintaining the phagocytic function of microglia
Source: CNS Neurosci Ther. 2023 Apr 18;29(10):2826–42. doi: 10.1111/cns.14219 (PMC10493665; doi:10.1111/cns.14219)
Supplement: Supplementary file 3 — Data S1 [file CNS-29-2826-s001.docx]

Degradation of NLRP3 by p62-dependent-autophagy improves cognitive function in Alzheimer's disease by maintaining the phagocytic function of microglia

**1. Materials and Methods**

**1.1 Immunostaining**

Cells were seeded into 6-well glass slide chambers at 2 x 10^5^ cells per well, cultured according to the experimental conditions, fixed with 4% paraformaldehyde for 15 minutes at room temperature, and permeabilized with PBS containing 0.5% Triton X-100. After blocking with PBS containing goat serum, the slides were incubated with primary antibody overnight at 4°C. The unbound antibody was removed by washing with PBS, and the slides were incubated with the appropriate secondary antibody and then washed. Nuclei were stained with aqueous DAPI (ab104139, abcam), and fluorescence was detected using a confocal microscope (Leica SP8 DIVE). Hemispheres of mouse brains were fixed in 10% formalin solution, and 3-µm-thick sagittal sections were cut, stained with antibodies, and stored overnight at 4°C. Specific test kits were used to identify the antigens. DAB was used for 60 s to develop the images, which were analyzed using Image J software. All positive depositions were analyzed in the hippocampus obtained from three random sections per brain.

**1.2 Co-IP and western blots**

Total protein was extracted from activated BV2 cells with Pierce® IP lysis buffer, containing the EDTA-free Halt™ protease inhibitor cocktail and PMSF protease inhibitor. Protein concentration was measured with the Pierce™ BCA protein assay kit with BSA as the standard protein. For co-IP experiments, total protein extracts from cells were incubated with the appropriate antibody at room temperature, then precipitated with protein A/G-agarose beads. The immunoprecipitated proteins were separated by SDS-PAGE and identified by mass spectrometric analysis or western immunoblotting. For western blots, lysates were mixed with SDS-PAGE sample loading buffer, separated by SDS-PAGE on a 4%-20% polyacrylamide gradient gel, and blotted onto polyvinylidene fluoride membranes. The membranes were blocked with 5% dry skim milk or 5% bovine serum albumin in tris-buffered saline Tween 20. Antibodies were diluted in a blocking buffer and incubated overnight at 4°C. β-tubulin was used as an internal control. The membranes were probed with anti-rabbit or anti-mouse HRP-conjugated IgG, developed using the Clarity™ Western ECL substrate, and visualized using a Bio-Rad GelDox XR imaging system.

**1.3 Quantitative real-time PCR (****qRT-PCR)**

The mRNA level of NLRP3 and p62 was determined by qRT-PCR. The corresponding gene-specific primer pairs and probe sequences are listed in **(Sup. Table 3)**. Total RNA was extracted from cells using the RNeasy Mini Kit(73404, Qiagen, China). Each sample was reverse transcribed to first-strand cDNA using 1 µg of total RNA and the PrimeScript™ RT reagent kit(18080051, Thermo) with gDNA Eraser. For each quantitative PCR reaction, 0.5% of the entire cDNA yield was used. Each reaction was performed using 2 µl of cDNA, optimized amounts of forward and reverse primers, and optimized amounts of TaqMan® Universal PCR Master Mix(7006437, Ranrun Jikang Biotechnology Co., Ltd, China). PCR tested the specificity of the primer, and their products were verified by sequencing provided by the sequencing platform of Ranrun Jikang Biotechnology Co., Ltd. The total mRNA levels were expressed as thousands of mRNA copies/μg total RNA. StepOnePlus(Thermo) was used in the qRT-PCR experiment.

**1.4 The method of the Mass Spectrometry analysis process**

In this study, the adhesive strip protein qualitative analysis technology was used to collect the protein samples prepared by SDS electrophoresis, and the Q ExactiveTM hf-x mass spectrometer was used for analysis to obtain proteomics information. The specific process is as follows.


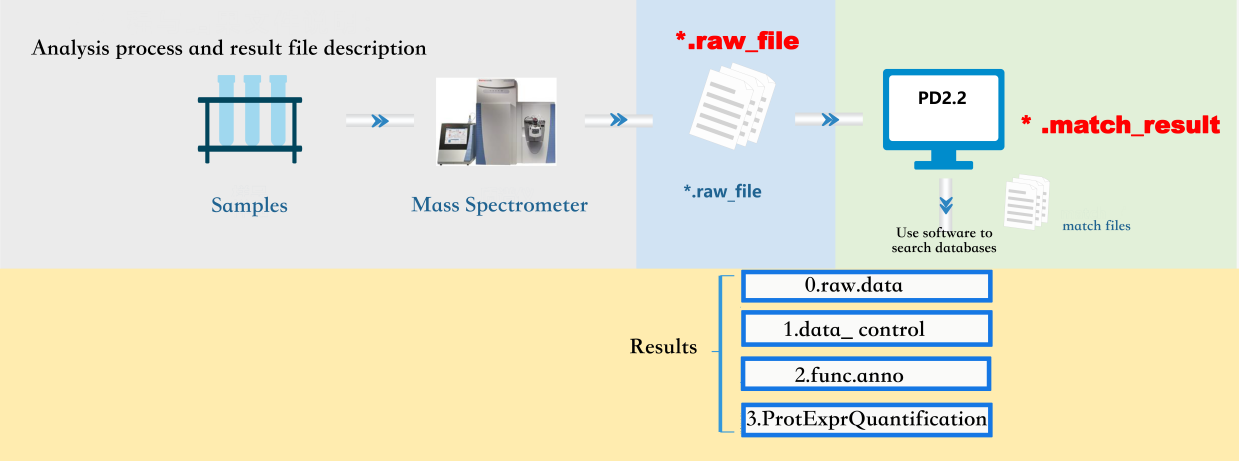


**1.4.1 Sample collection and processing**

Protein samples that may interact with NLRP3 were collected from LPS+ Aβ 1-42-stimulated BV2 cells by the co-IP method. (Materials and Methods 2.7) The collected protein complex samples were separated by SDS-PAGE electrophoresis. When the length of running glue was 1cm, the electrophoresis was stopped, and the tape samples containing protein were collected. The tape was stained with Coombe bright blue. The cut gel was destained by 50% acetonitrile (ACN) in 50 mM triethylammonium bicarbonate (TEAB) and dehydrated upon washing with 100% ACN till the gel turned white. Proteins in the gel were treated with 1000 µL 10 mM DTT for 40 min at 56°C and subsequently alkylated with 1000 µL 50mM IAM for 30 min in the dark. The gel was then washed with destained buffer and treated with ACN as above.

**1.4.2 In-gel Digestion**

10-20 μL 10 ng/µL trypsin was added into gel and incubation for 30 min on ice, then made up to 100 μL with 100 mM TEAB. Proteins were digested overnight at 37°C. After centrifugation at low speed, the supernatant was collected, and the remaining peptides were extracted in 100 μL of 0.1% formic acid (FA). Combined the two supernatant and centrifuged at 12000 g for 5 min at room temperature. The supernatant was slowly loaded to the C18 desalting column, washed with 1 mL of washing solution (0.1% formic acid, 4% acetonitrile) 3 times, then eluted twice by 0.4 mL of elution buffer (0.1% formic acid, 75% acetonitrile). The eluents were combined and lyophilized.

**1.4.3 LC-MS/MS Analysis**

Mobile phase A (100% water, 0.1% formic acid) and B solution (80% acetonitrile, 0.1% formic acid) were prepared. The lyophilized powder was dissolved in 10 μL of solution A, centrifuged at

15,000 rpm for 20 min at 4℃and 1 μg of the sample was injected into a home-made C18 Nano-Trapcolumn (2 cm×75 μm, 3 μm). Peptides were separated in a homemade analytical column (15 cm×150 μm, 1.9 μm), using a linear gradient elution as listed in Table 1. The separated peptides were analyzed by Q Exactive HF-X mass spectrometer (Thermo Fisher), with an ion source of Nanospray Flex™（ESI）, spray voltage of 2.3 Kv, and ion transport capillary temperature of 320°C. Full scan range from m/z 350 to 1500 with a resolution of 60000 (at m/z 200), an automatic gain control (AGC) target value was 3×10 6 and a maximum ion injection time was 20 ms. The top 40 precursors of the highest abundant in the full scan were selected and fragmented by higher-energy collisional dissociation (HCD) and analyzed in MS/MS, where the resolution was 15000 (at m/z 200), the automatic gain control (AGC) target value was 1×10 5, the maximum ion injection time was 45 ms, normalized collision energy was set as 27and an intensity threshold was 2.2×10 4, and the dynamic exclusion parameter was 20 s. The raw data of MS detection was named “.raw”.

**1.4.4 The identification of protein**

The resulting spectra from each fraction were searched separately against Mus_musculus_uniprot_2019.01.18.fasta (85188 sequences) database by the search engines: Proteome Discoverer 2.2 (PD 2.2, Thermo). The search parameters are set as follows: mass tolerance for precursor ion was 10 ppm and mass tolerance for production was 0.02 Da. Carbamidomethyl was specified in PD 2.2 as fixed modifications. Oxidation of methionine (M) and acetylation of the N-terminus was specified in PD 2.2 as variable modifications. A maximum of 2 missed cleavage sites were allowed.

The identified protein contains at least 1 unique peptide with an FDR of no more than 1.0%. Proteins containing similar peptides that could not be distinguished by MS/MS analysis were identified as the same protein group.

**1.4.5 The functional analysis of protein**

Gene Ontology (GO) was conducted using the interproscan-5 program against the non-redundant protein database (including Pfam, PRINTS, ProDom, SMART, ProSiteProfiles, PANTHER) [1], KEGG (Kyoto Encyclopedia of Genes and Genomes) were used to analyze the protein family and pathway. KEGG and COG: 1) KEGG and COG annotation is a BLAST comparison of identified proteins (BLASTP, EVALue ≤ 1E-4); 2) BLAST result filtering: For BLAST results of each sequence, the comparison results with the highest score are selected for annotation.

**1.4.6Criteria and procedures for screening target proteins**

**1.4.6.1** Non-specific proteins identified in the IgG control group and LPS treated group were filtered out.

**1.4.6.2** The screened proteins were classified according to function, including

1. Target proteins involved in protein regulation
2. Myosin related protein
3. Uncharacterized protein
4. ribosomal protein
5. cytoskeletal related protein
6. Other proteins

The results of the arrangement are in the MS results.xlsx in the supplementary material(Results of mass spectrometry).zip

**Reference**

[1] Jones P, Binns D, Chang H Y, et al. InterProScan 5: genome-scale protein function

classification[J]. Bioinformatics, 2014, 30(9): 1236-1240.

**1.5 RNA-seq analysis**

**
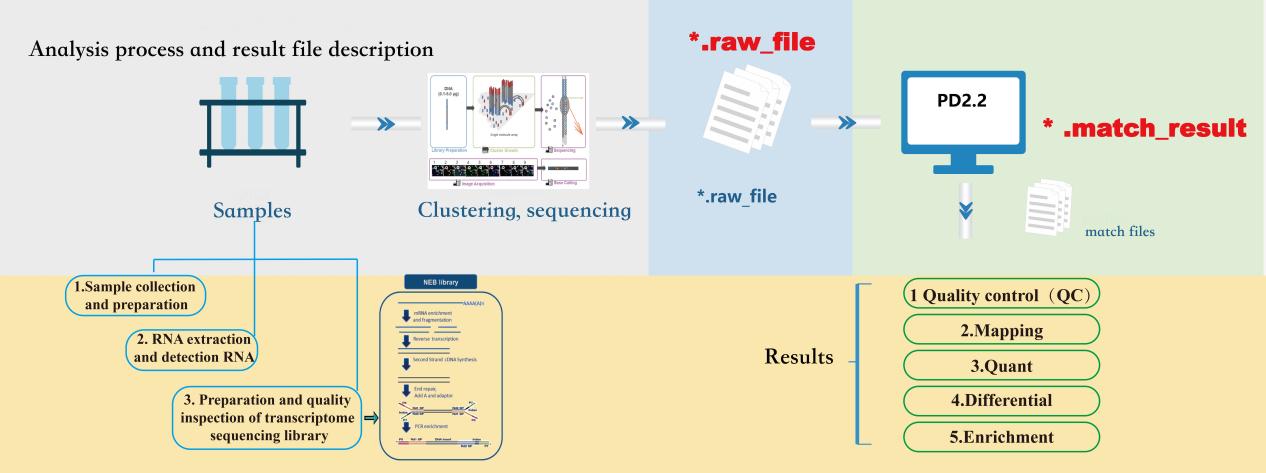
**

**1.5.1. Sample collection and preparation**

Sample collection: Add 1 ml TRIzol reagent to each well in the six-well plate, blow the cells with a pipette repeatedly, fully digest the cells, transfer them to an RNase-free 1.5 mL centrifuge tube, and suction them repeatedly with a disposable syringe until no clumps of cells can be seen. The whole solution is clear but not sticky. Store in refrigerator at -80℃.

**1.5.2. RNA extraction and detection of RNA**

Cellular RNA was extracted using standard extraction methods. RNA integrity was assessed using the RNA Nano 6000 Assay Kit of the Bioanalyzer 2100 system (Agilent Technologies, CA, USA).

**1.5.3. Preparation and quality inspection of transcriptome sequencing library**

The starting RNA for library construction was total RNA (total RNA was no less than 1ug), using fragment mRNA as a template. Using divalent cations under elevated temperature, fragmentation was carried out in First Strand Synthesis Reaction Buffer(5X). First-strand cDNA was synthesized using random hexamer primer and M-MuLV Reverse Transcriptase, then use RNaseH to degrade the RNA. Second strand cDNA synthesis was subsequently performed using DNA Polymerase I and dNTP. The remaining overhangs were converted into blunt ends via exonuclease/polymerase activities. After adenylation of 3’ ends of DNA fragments, Adaptor with hairpin loop structure was ligated to prepare for hybridization. To select cDNA fragments of preferentially 370~420 bp in length, the library fragments were purified with AMPure XP system (Beckman Coulter, Beverly, USA). Then PCR was performed with Phusion High-Fidelity DNA polymerase, Universal PCR primers, and Index (X) Primer. At last, PCR products were purified (AMPure XP system) and library quality was assessed on the Agilent Bioanalyzer 2100 system. The effective concentration of the library is higher than 2nM to ensure library quality and complete library quality inspection

**1.5.4. Clustering, sequencing**

The clustering of the index-coded samples was performed on a cBot Cluster Generation System using TruSeq PE Cluster Kit v3-cBot-HS (Illumia) according to the manufacturer’s instructions. After cluster generation, the library preparations were sequenced on an Illumina Novaseq platform and 150 bp paired-end reads were generated.

**1.5.5. Data Analysis**

**1.5.5.1 Quality control**

Raw data (raw reads) of fastq format were firstly processed through in-house perl scripts. In this step, clean data (clean reads) were obtained by removing reads containing adapter, reads containing N base and low-quality reads from raw data. At the same time, Q20, Q30, and GC content of the clean data were calculated. All the downstream analyses were based on the clean data with high quality.

**1.5.5.2. Reads mapping to the reference genome**

Reference genome and gene model annotation files were downloaded from the genome website directly. Index of the reference genome was built using Hisat2 v2.0.5 and paired-end clean reads were aligned to the reference genome using Hisat2 v2.0.5. We selected Hisat2 as the mapping tool that Hisat2 can generate a database of splice junctions based on the gene model annotation file and thus a better mapping result than other non-splice mapping tools.

**1.5.5.3. Quantification of gene expression level**

FeatureCounts v1.5.0-p3 was used to count the reads numbers mapped to each gene. And then FPKM of each gene was calculated based on the length of the gene and the reads count mapped to this gene. FPKM, the expected number of Fragments Per Kilobase of transcript sequence per Millions of base pairs sequenced, considers the effect of sequencing depth and gene length for the reads count at the same time, and is currently the most used method for estimating gene expression levels.

**1.5.6. Bioinformatics analysis**

1.5.6.1 Differential expression analysis

(For DESeq2 with biological replicates) Differential expression analysis of two conditions/groups (two biological replicates per condition) was performed using the DESeq2 R package (1.20.0). DESeq2 provides statistical routines for determining differential expression in digital gene expression data using a model based on the negative binomial distribution. The resulting P-values were adjusted using Benjamini and Hochberg’s approach for controlling the false discovery rate. Genes with an adjusted P-value <0.05 found by DESeq2 were assigned as differentially expressed.

(For edgeR without biological replicates) Prior to differential gene expression analysis, for each sequenced library, the read counts were adjusted by the edgeR program package through one scaling normalized factor. Differential expression analysis of two conditions was performed using the edgeR R package (3.22.5). The P values were adjusted using the Benjamini & Hochberg method. Corrected P-value of 0.05 and absolute foldchange of 2 was set as the threshold for significantly differential expression.

1.5.6.2 Enrichment analysis of differentially expressed genes （KEGG GO）

Gene Ontology (GO) enrichment analysis of differentially expressed genes was implemented by the clusterProfiler R package, in which gene length bias was corrected. GO terms with an adjusted P-value(q-value) less than 0.05 were considered significantly enriched by differential expressed genes. KEGG is a database resource for understanding high-level functions and utilities of the biological system, such as the cell, the organism, and the ecosystem, from molecular-level information, especially large-scale molecular datasets generated by genome sequencing and other high-throughput experimental technologies (http://www.genome.jp/kegg/). We used the clusterProfiler R package to test the statistical enrichment of differential expression genes in KEGG pathways. The Reactome database brings together the various reactions and biological pathways of human model species. Reactome pathways with corrected P-value less than 0.05 were considered significantly enriched by differential expressed genes. The DO (Disease Ontology) database describes the function of human genes and diseases. DO pathways with corrected P-value less than 0.05 were considered significantly enriched by differential expressed genes. The DisGeNET database integrates human disease-related genes. DisGeNET pathways with corrected P-value less than 0.05 were considered significantly enriched by differential expressed genes. We used clusterProfiler software to test the statistical enrichment of differentially expressed genes in the Reactome pathway, the DO pathway, and the DisGeNET pathway.

1.5.6.3 KEGG pathway in the target pathway-related molecular expression

Molecular difference analysis was performed for pathways with high correlation in KEGG pathway analysis results, and heat map display was performed with RStudio software.

**1.6 Mouse behavioral tests**

**1.6.1 Open-field test**

The open-field test(OFT) is a method to evaluate the autonomous behavior, inquiry behavior, and anxiety of experimental animals in a new environment[29]. The experiment apparatus is a 50 cm x 50 cm x30 cm white box with no top. The mice were placed in the center of the box, and their behavior was automatically recorded for 5 minutes.

The decrease in velocity, frequency across the center area (C zone), and moving distance in the center zone indicated that the group was less curious about new things and was in line with anxiety and depression behavior. In addition, animals with high anxiety levels tended to spend less time in the central zone and longer *not moving cumulative duration*. Other details are described in the previous article[30].

**1.6.2 Novel object recognition test**

The novel object recognition (NOR) test assessed short- and long-term recognition memory [31]. The experimental procedure includes three stages. The 1st day was the adaptation period with no toy bricks. The 2nd day was the familiarization period with two toy bricks (2 cm x 2 cm) with the same shape, size, and color in opposite corners. The 3rd day was the test period with one of the toy bricks changed to a different shape and color. The camera on the top of the box was used to record how long a mouse took to examine the new object (TN) and the familiar object (TF), allowing five minutes for exploring the two different objects. We used a recognition index and a preference index to indicate recognition memory. Other details were described in the previous article[30].

Discrimination index = (TN-TF) / (TN+TF)×100%,

Preference index = TN/ (TN+TF)×100%

The higher recognition index and the preference index reflect better short- and long-term recognition memory ability of animals in this group.

**1.6.3 The Morris water maze**

The Morris water maze (MWM), consisting of spatial training trials and probe trials[32-34], evaluated hippocampus-dependent spatial navigation learning and memory. The water maze is divided into four quadrants and contains a platform that stays under 1-1.5 cm of water. The mice were placed in the water from the opposite quadrant of the platform and were deemed successful in finding the platform. During the training trials, the mouse was given three trial sessions each day for four or five consecutive days (according to how long the WT group takes to reach over a 95% successful rate). If the mice did not find the platform within 1 minute, the latency to the platform was recorded as 60 seconds. Other details have been described in previous studies[35].

Spatial training trials: longer latency to the platform indicating poor memory and learning ability.

Probe trials: Less frequency to the platform, shorter time in the platform quadrant, shorter cumulative duration in the target zone, and longer latency to the platform indicating poor memory.

**1.6.4 The Y-maze test**

In the Y-maze test, each mouse was placed at the end of one arm of the ‘Y’ and allowed to explore the maze freely for five minutes. The sequence of entry of the mice in the arms was recorded to calculate the percentage of change. Spontaneous alternations (%) = number of alternations (N) / (N-2) x100%. Other details are described in the published procedure[23].

The higher spontaneous alternations indicated better learning and memory ability.

References are in the **main document.**

**1.7 Supplementary material: experimental animals**

NLRP3 knockout mice with a C57BL/6 background were constructed using the CRISPR/Cas9 genome editing technique. This gene has three transcripts according to the Ensembl website (http://asia.ensembl.org/Mus_musculus/Gene/Summary?g=ENSMUSG00000032691;r=11:59541568-59566956). In this study, the transcript NLRP3-202 was used for gene editing. Guide-RNA was designed for exon 2 of mouse NLRP3 gene transcript 202. The specific information of the gRNA is GAAGATTACCGCCCGAGAA. gRNA was synthesized in vitro, transcribed, and injected into C57BL/6 mouse fertilized ovum cells together with Cas9 RNA. The fertilized ovum cells were transplanted into donor ICR mice. After birth, genomic DNA was extracted from the tails of the offspring, and the target genes were amplified by PCR. Genotypes were identified after sequencing. A deletion mutation of 12 bp was identified by sequencing, and mice with an insertion of 1 bp (△12Ins1) and a deletion mutation of 11 bp (△11) were identified. We generated mice with a deletion mutation of 12 bp and an insertion of 1 bp (△12Ins1) identified by sequencing mice with an 11 bp deletion mutation (△11)

| NO. | WT | NKRP3KO |
| --- | --- | --- |
| 1#（♂） | TACCCGCCCGAGAAAGGCTGTAT | TAC------------GGCTGTAT  T |
| 2#（♂） | CGCCCGAGAAAGGCTGTATCCCA | CGC-----------TGTATCCCA |

**1.8 Plasmid build**

**1.8.1 pLVX-mNlrp3-3HA-Puro**

Gene Name：mouse Nlrp3 ((NM_ 145827.4))

Cloning Vector：pLVX-3HA-PGK-Puro

Cloning Strategy：XhoI+BamHI

Vector map：


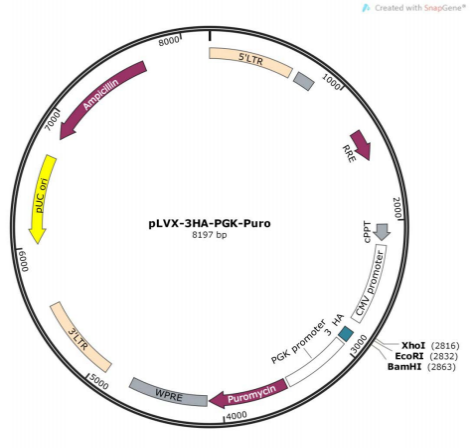
mouse Nlrp3 sequence：3117bp

**1.8.2 pLVX-mP62-3flag-Hygro**

Construct Information:

Gene Name: mouse P62 (NM_011018.3)

Cloning Vector: pLVX-EF1a-SV40-Hygro

Cloning Strategy: EcoRI+AgeI

Vector Map:


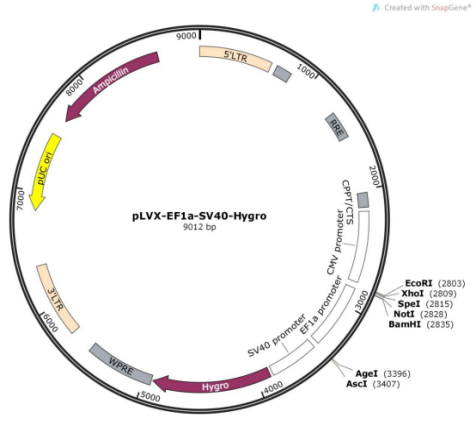


mouse P62 sequence：1413bp

**1.8.3 pLVX-shRNA1-mNlrp3/mP62**

Construct Information:

Gene Name:

mouse Nlrp3 (GeneID:216799)

mouse P62 (GeneID:18412)

Cloning Vector: pLVX-shRNA1

Cloning Strategy: BamHI+EcoRI


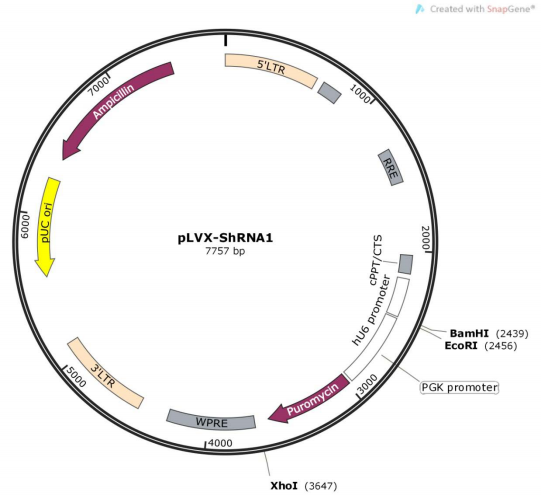
Vector Map:

mNlrp3-1 sequence：5‘-GGATCTTTGCTGCGATCAACA-3’

mP62-3 sequence：5‘-GGACCCATCTACAGGTGAACT-3’

**1.9 Download address of MS results.zip and RNA-seq analysis.zip**

RNA-seq results:

https://1drv.ms/u/s!AmfTzIfNgm35gS2RzmC-6QvqCXN9?e=CEimIk

MS. results:

https://1drv.ms/u/s!AmfTzIfNgm35gSx6HWjvhckVX_vX?e=NNo4Ke

**Supplementary Table 1**

Table 1. Reagents and antibodies.

| Reagents and antibodies | Company | Product code | Final Concentration | Reagents and antibodies | Company | Product code | Final Concentration |
| --- | --- | --- | --- | --- | --- | --- | --- |
| LPS | Solarbio | L8880 | 100ng/ml | Mouse monoclonal to SQSTM1 / p62 (IF) | Abcam | ab56416 | 1:100 |
| B-Amyloid(1-42), Oligomers, Human | fooyue Biotechnology (Shanghai) Co., Ltd | 45434534 | 10μM | anti-NLRP3 antibody（WB） | Abcam | ab210491 | 1:1000 |
| RhodamineB-Amyloid (1-42), Oligomers, Human | fooyue Biotechnology (Shanghai) Co., Ltd | 77232321 | 10μM | Mouse monoclonal to SQSTM1 / p62 (WB) | Abcam | ab56416 | 1:1000 |
| 3-Methyladenine-autophagy(3-MA) | Sigma | M9281-100MG | 5mM | anti-LAMP-1 antibody (WB) | Abcam | ab208943 | 1:1000 |
| MG-132 (Ethanol Solution)， a proteasome inhibitor | Abcam | ab147047 | 10μM | anti-Beclin-1 antibody (WB) | CST | 3495T | 1:1000 |
| Puromycin | Solarbio | P8230 | 5μg/ml | anti-LC3A/B antibody (WB) | CST | 12741T | 1:1000 |
| Hygromycin B | Solarbio | H8080 | 200ug/ml | anti-mTOR antibody (WB) | CST | 2983T | 1:1000 |
| Blasticidin S | Solarbio | B9300 | 5μg/ml | anti-p-mTOR antibody (WB) | CST | 5536T | 1:1000 |
| CD16/CD32， P E-Cyanine7 | eBioscience™ | 25-0161-81 | 1.25μl/test | anti-AKT antibody (WB) | CST | 4691T | 1:2000 |
| Rat IgG2a kappa Isotype Control， E-Cyanine7 | eBioscience™ | 25-4321-81 | 1.25μl/test | anti-pAKT antibody (WB) | CST | 4060T | 1:2000 |
| CD86 (B7-2), FITC | eBioscience™ | 11-0862-81 | 0.25μl/test | Anti-Ubiquitin (linkage-specific K63) antibody (WB) | Abcam | ab179434 | 1:1000 |
| Rat IgG2a kappa Isotype Control， FITC | eBioscience™ | 11-4321-82 | 0.25μl/test | HRP Anti-beta Tubulin antibody | Abcam | ab21058 | 1:1000 |
| CD86 (B7-2), PerCP-eFluor 710 | eBioscience™ | 46-0862-80 | 0.25μl/test | Goat Anti-Rabbit IgG H&L (HRP) | ZSGB-BIO | ZB-2201 | 1:5000 |
| Rat IgG2a kappa Isotype Control, PerCP-eFluor 710 | eBioscience™ | 46-4321-82 | 0.25μl/test | Goat Anti-mouse IgG H&L (HRP) | ZSGB-BIO | ZB-2305 | 1:5000 |
| NLRP3 (D4D8T) Rabbit mAb(co-IP,WB) | CST | 15101 | 1:200 | TRITC goat anti-mouse | EARTHOX | 920871 | 1:200 |
| SQSTM1/p62 Rabbit mAb (co-IP) | CST | 23214 | 1:200 | IITC goat anti-rabbit | EARTHOX | 220591 | 1:200 |
| HA-Tag Rabbit mAb(co-IP) | CST | 3724s | 1:50 | anti-IBA-1 antibody（IHC） | Abcam | ab178846 | 1:2000 |
| DYKDDDDK Tag Rabbit mAb(anti-FLAG)(co-IP) | CST | 14793s | 1:50 | anti-Aβ antibody（IHC） | Abcam | ab201060 | 1:1000 |
| DYKDDDDK Tag Mouse mAb(anti-Flag)(WB) | CST | 8146T | 1:1000 | Immunohistochemical test kit | ZSGB-BIO | PV-6001 |  |
| Rabbit mAb IgG Isotype Control | CST | 3900 | 5ul/1ml | anti-NLRP3 antibody（IF） | Abcam | ab4207 | 1:100 |
| Iba-1 antibody(IF) | WAKO | 019-19741 | 1:1000 | IL-1β Elisa kit | Elabscience | E-el-M0037c |  |

**Supplementary Table 2**

Table 2. Primers are used for genotype identification.

| GENES | Forward primers | Reverse primers |
| --- | --- | --- |
| 5×FAD | AGGACTGACCACTCGACCAG | CGGGGGTCTAGTTCTGCAT |
| NLRP3-KO | ACCTGTACCAATCTATGGGGT | TTATGTCACGTGTACCTTACTGT |

**Supplementary Table 3**

Table 3. Primers used for qRT-PCR.

| GENES | Forward primers | Reverse primers | Primers for fluorescence probe |
| --- | --- | --- | --- |
| NLRP3 | TCTACTCTATCAAGGACAGGAACGC | CTCCTGCTTGCTTGGATGCT | TACACGCAGCTCCAACT(5'FAM3'MGB) |
| GAPDH | GCACAGTCAAGGCCGAGAA | CCTCACCCCATTTGATGTTAGTG | TCTTCCAGGAGCGAGAC(5'FAM3'MGB) |

**Supplementary Figure Legend**

**Supplementary Figure 1. NLRP3 inflammasome activation in the *in vivo* and *in vitro* AD models. (a, c, e, f)** The NLRP3, pro-Caspase1, caspase 1-p10 expression levels *in vivo*. **(b, d, g, h)** The NLRP3, pro-Caspase1, caspase 1-p10 expression levels *in vitro*. **(i)** Expression of IL-1β in supernatants of different drug-treated BV2 cells by ELISA. **(j)** Statistical analysis of the diameter of AB plaques in the hippocampus and frontal cortex of 9.5-month-old mice. Data are reported as mean ± SEM (n = 3) of individual experiments.*= p<0.05, **= p<0.01, **** p=<0.0001, #= p<0.05, ##= p<0.01, ###= p<0.001, ####= p<0.0001.

**Supplementary Figure 2. RNA-seq analysis of *in vitro* AD model vs LPS treated BV2 cells.**

1. Differential gene analysis comparing BV2 cells treated with LPS plus Aβ1-42 and cells treated with LPS only.
2. GO enrichment analysis of the differentially up-regulated genes is shown in the **Fig. S2a** (the most significant 30 up-regulated genes terms were selected to draw a scatter diagram for display, padj<0.05).
3. GO enrichment analysis of differentially down-regulated genes is shown in the **Fig. S2a**(the most significant 30 down-regulated genes terms were selected to draw a scatter diagram for display, padj<0.05). .
4. KEGG pathway analysis of differentially expressed genes in the **Fig. S2a**(the most significant 20 terms were selected to draw a scatter diagram for display, padj<0.05) in Fig. S2a.

BP: biological processes, CC: cellular components, MF: molecular functions.
