# Supplementary material for: Degradation of NLRP3 by p62‐dependent‐autophagy improves cognitive function in Alzheimer's disease by maintaining the phagocytic function of microglia
Source: CNS Neurosci Ther. 2023 Apr 18;29(10):2826–42. doi: 10.1111/cns.14219 (PMC10493665; doi:10.1111/cns.14219)

Figure 5a

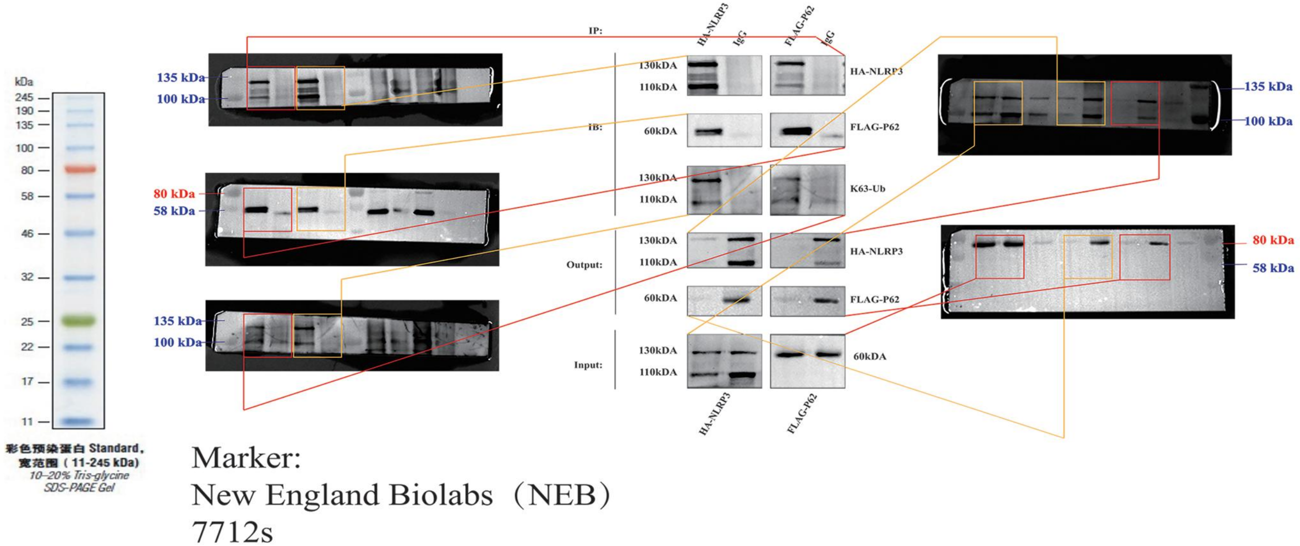

Marker:
New England Biolabs（NEB）
7712s

Figure 5b

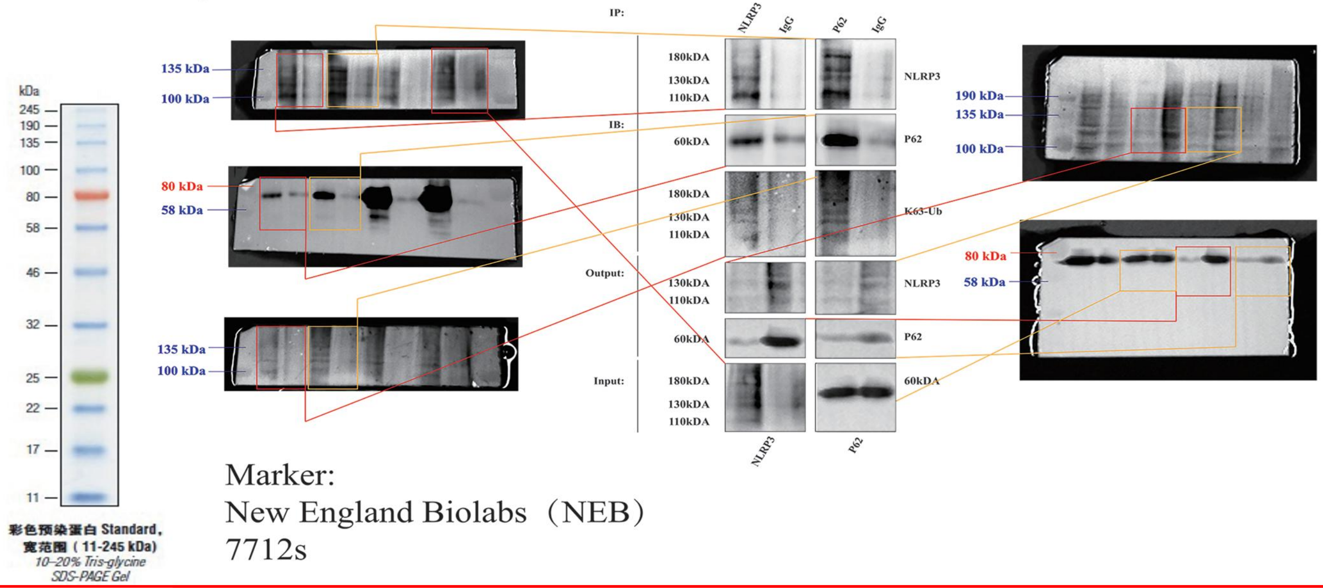

Marker:
New England Biolabs（NEB）
7712s

Figure 5d

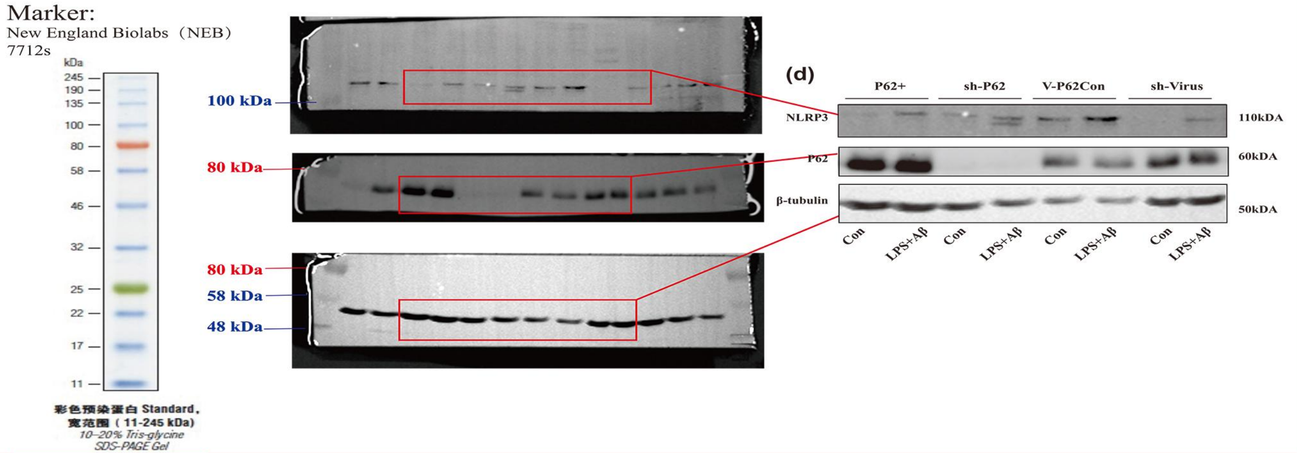

Figure 5e

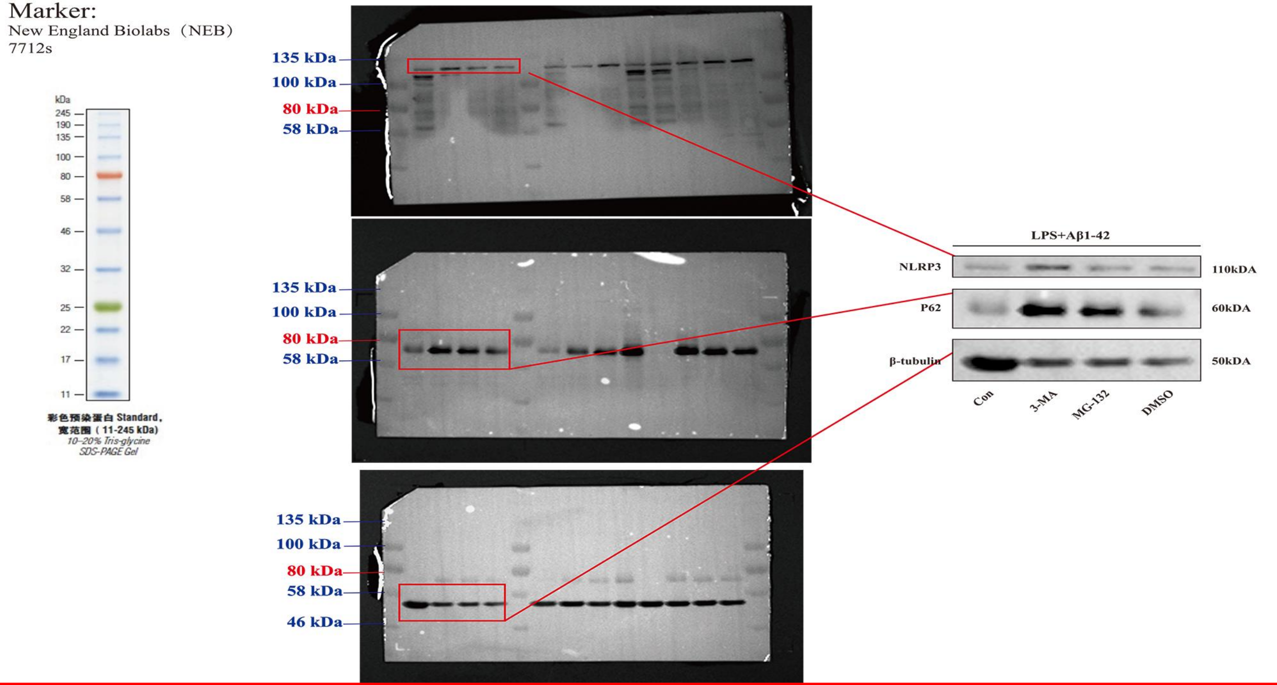

Figure 7b

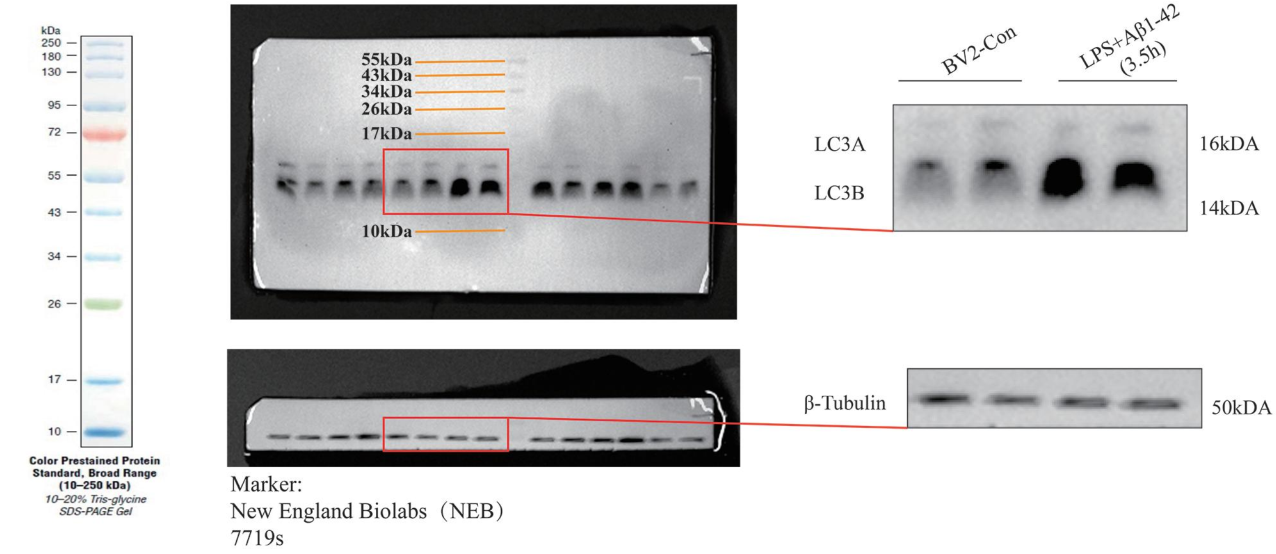

Figure 7c

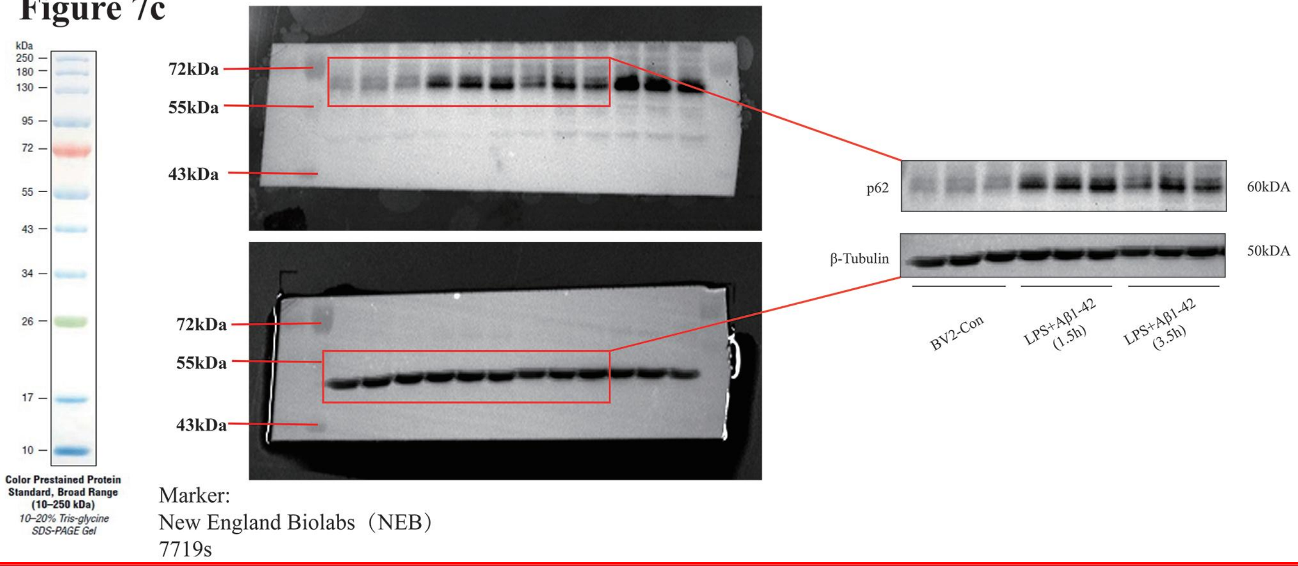

Fig. S1A

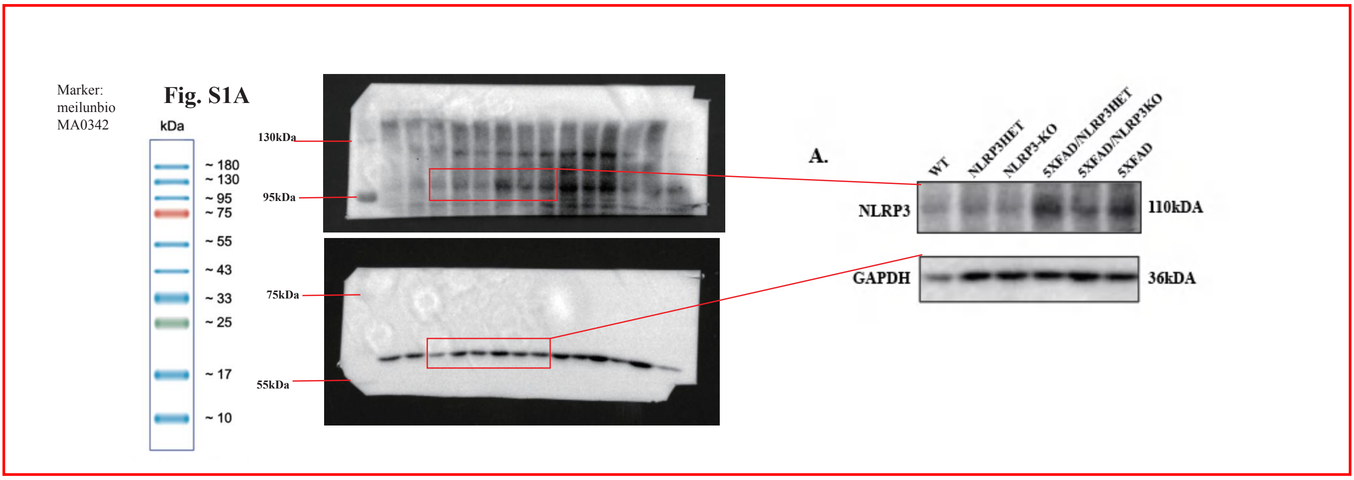

Fig. S1E

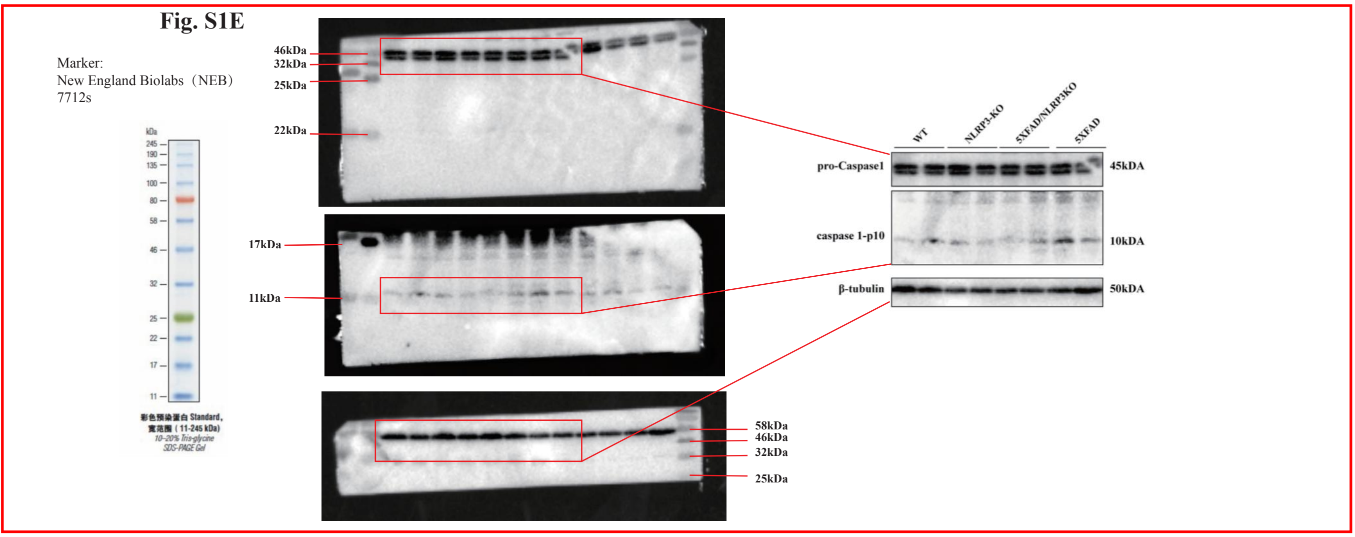

Fig. S1B

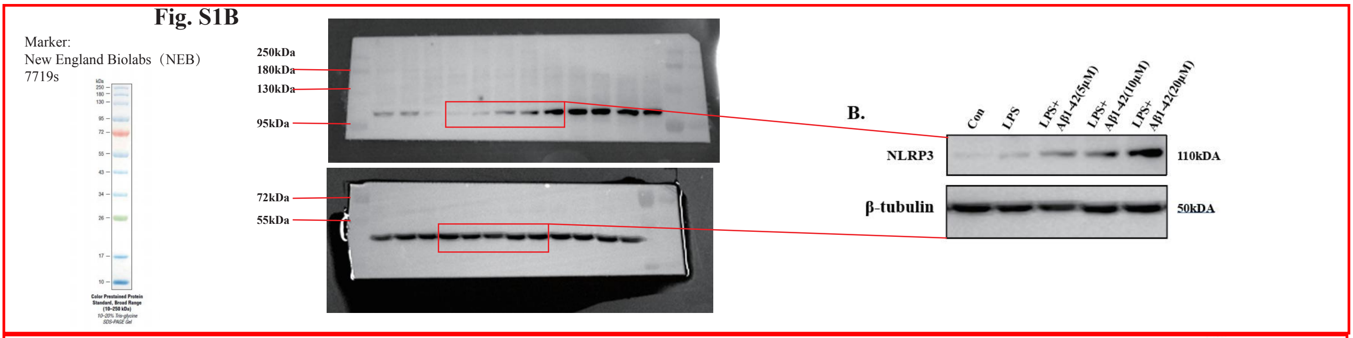

Fig. S1G

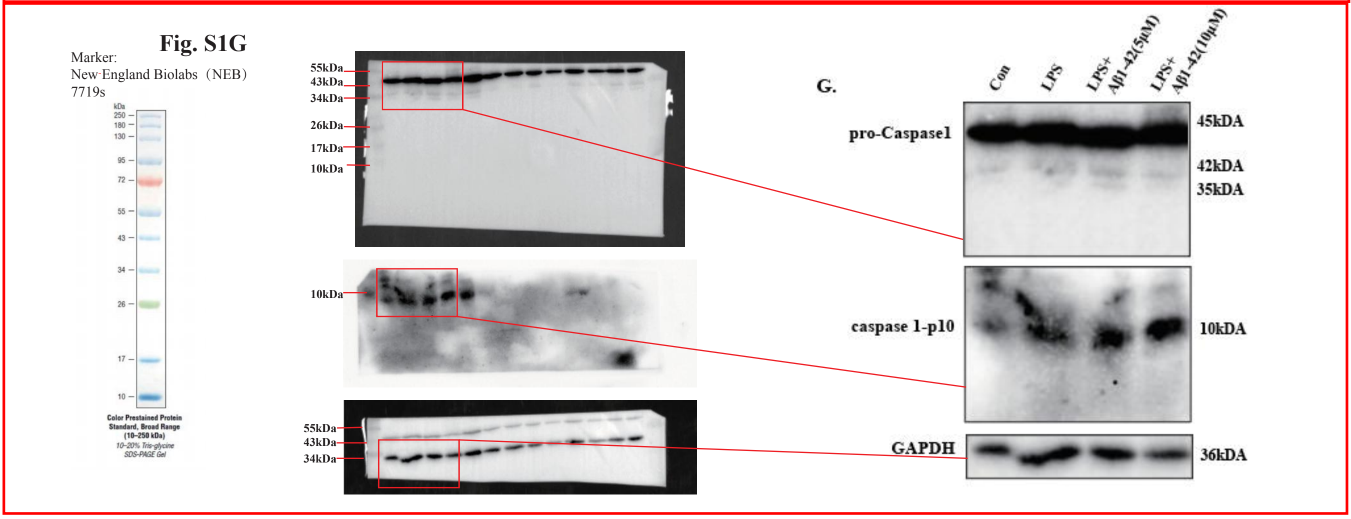

Supplement: Supplementary file 5 — Data S3 [file CNS-29-2826-s004.pdf]
